# Microwave-Assisted Hydrothermal Synthesis of Photocatalytic Truncated-Bipyramidal TiO_2_/Ti_3_CN Heterostructures Derived from Ti_3_CN MXene

**DOI:** 10.1021/acs.langmuir.4c02444

**Published:** 2024-10-04

**Authors:** Muhammad Abiyyu Kenichi Purbayanto, Madhurya Chandel, Dominika Bury, Anna Wójcik, Dorota Moszczyńska, Anika Tabassum, Vadym N. Mochalin, Michael Naguib, Agnieszka Maria Jastrzębska

**Affiliations:** † Faculty of Materials Science and Engineering, Warsaw University of Technology, Wołoska 141, Warsaw 02-507, Poland; ‡ Faculty of Mechatronics, Warsaw University of Technology, św. Andrzeja Boboli 8, Warsaw 02-525, Poland; § Polish Academy of Sciences, Institute of Metallurgy and Materials Science, W. Reymonta 25, Cracow 30-059, Poland; ∥ Department of Physics and Engineering Physics, Tulane University, New Orleans, Louisiana 70118, United States; ⊥ Department of Chemistry, Missouri University of Science and Technology, Rolla, Missouri 65409, United States; # Department of Materials Science and Engineering, Missouri University of Science and Technology, Rolla, Missouri 65409, United States

## Abstract

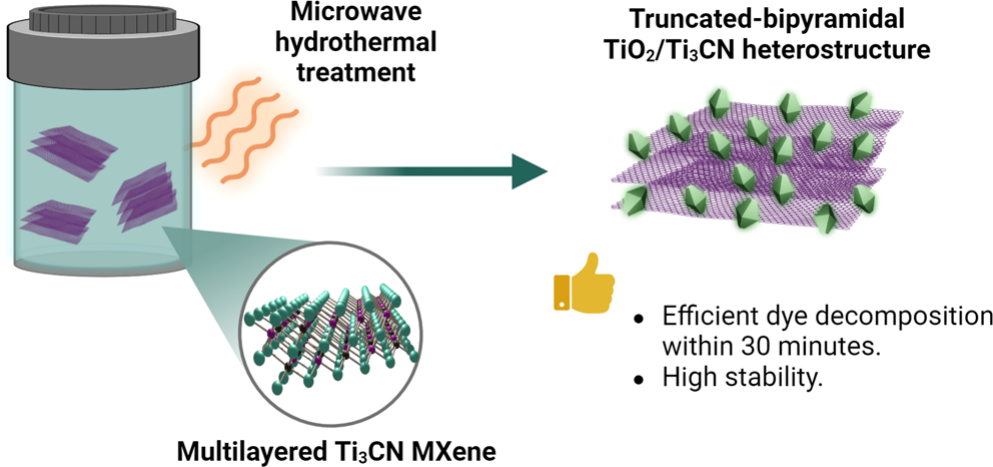

TiO_2_/MXene heterostructure has garnered significant interest as a photocatalyst due to its large surface area and efficient charge carrier separation at the interface. However, current synthesis methods produce TiO_2_ without clear crystal faceting and often require complicated postprocessing step, limiting its practical applications. We demonstrate a facile and controlled microwave-assisted hydrothermal synthesis for transforming multilayered Ti_3_CN MXene to a truncated-bipyramidal TiO_2_/Ti_3_CN heterostructure. The resulting TiO_2_ nanocrystals at the Ti_3_CN surface exhibited crystalline anatase truncated bipyramids, exposing {001} and {101} facets. We further tailored an indirect optical band gap of the TiO_2_/Ti_3_CN heterostructure in the range of 3.17–3.23 eV by varying the hydrothermal synthesis time from 15 min to 5 h at a fixed temperature of 160 °C. Efficient charge separation allowed us to decompose 97% of methylene blue (MB) within 30 min of ultraviolet (UV) light irradiation, ∼3.9-fold faster than the benchmark P25, higher than any other TiO_2_/MXene heterostructures. With simulated white light, we achieved over 60% efficiency of the dye decomposition within 2 h of irradiation, which resulted in 1.5-fold faster kinetics than P25. We also observed a similar excellent performance of Ti_3_CN-derived TiO_2_ in decomposing various persistent synthetic dyes, including commercial textile dye, methyl orange, and rhodamine B. In conclusion, our study provides a strategy for utilizing MXene chemical reactivity to produce highly crystalline optically active TiO_2_/Ti_3_CN heterostructure. The developed heterostructure can serve as an efficient photocatalyst for the degradation of organic pollutants.

## Introduction

The urgency of addressing environmental concerns has underscored the need for the development of efficient and sustainable photocatalytic materials capable of degrading organic pollutants in wastewater.^1−3^ Anatase titanium dioxide (TiO_2_) has garnered extensive attention and practical application as a photocatalyst owing to its notable catalytic activity and low cost.^4^ However, anatase TiO_2_ has a large band gap of 3.2 eV (which corresponds to ultraviolet (UV) light energy and translates into inefficient utilization of the larger fraction of sunlight), as well as rapid electron and hole recombination, leading to the inability to separate photogenerated charges effectively.^5^ Therefore, researchers implemented doping, heterostructure, and crystal facet engineering to improve the photocatalytic performance of TiO_2_.^6,7^

In particular, heterostructure engineering is considered one of the most promising methods for preparing advanced photocatalysts due to its effectiveness in achieving spatial separation of electron–hole pairs.^8^ Furthermore, incorporating heterojunctions with cocatalysts can prolong the charge carrier lifetime in semiconductor photocatalysts, significantly enhancing their photocatalytic activity.^9,10^ The discovery of two-dimensional (2D) materials opened the possibility of obtaining hybrid photocatalysts with a large specific surface area, high charge carrier mobility, and high photostability.^8^ Various 2D materials, including graphene, g-C_3_N_4_, MoS_2_, and MXenes, have been coupled with TiO_2_ and showed remarkable photocatalytic performance.^11^

MXenes, a large family of 2D transition-metal carbides, nitrides, and carbonitrides, have been used as precursors or templates for obtaining TiO_2_ nanocrystals and TiO_2_-based heterostructure.^12,13^ Ti_3_C_2_ has shown promise as a precursor in obtaining the TiO_2_/Ti_3_C_2_ heterostructure with optical absorption strong enough to apply in photocatalysis.^10,14,15^ However, Ti_3_C_2_ MXenes produced TiO_2_ with poor crystallinity and no clear crystal faceting, leading to limited photocatalytic performance.^15−17^ Additionally, the synthesis of the TiO_2_/MXene heterostructure often requires high calcination temperatures and additional directing reagents, hindering its potential for practical applications.^14,17^

We posit that Ti_3_CN may serve as a compelling precursor, extending beyond commonly employed carbide-based MXenes. Based on the density functional theory (DFT) study, Ti_3_CN exhibits higher electrical conductivity than Ti_3_C_2_.^18^ Additionally, carbon and nitrogen atoms are dispersed randomly in the 2D Ti_3_CN crystal lattice, resulting in improved chemical reactivity.^19^ Prior experimental studies of MXene reactivity with water showed that Ti_3_CN displays a notably higher reactivity toward an aqueous environment when compared to Ti_2_C, Ti_3_C_2_, and Nb_2_C MXenes.^19^ This characteristic may result in Ti_3_CN being easier to be *in situ*-decorated by TiO_2_*via* hydrolysis or oxidation compared to the Ti_3_C_2_ counterpart.

In this study, by employing a facile approach that involves a microwave-assisted hydrothermal method, hydrogen peroxide (H_2_O_2_), and the exceptional reactivity of Ti_3_CN MXene, we have successfully obtained TiO_2_ decorating the surface of Ti_3_CN in the form of highly crystalline anatase truncated bipyramids, with exposed {001} and {101} facets. A well-defined crystalline phase of TiO_2_ ensures low electron transfer resistance and reduces recombination rates of photogenerated electrons and holes at the TiO_2_/MXene interface.^20^ Additionally, by optimizing the morphology of the nanomaterials, the specific surface area can be enhanced, leading to improved photocatalytic performance.^21^

We explore a range of oxidation durations, from 15 min to 5 h. The maximum oxidation duration was selected based on the previous study by Ahmed et al., in which reactive MXene (Ti_2_C) is completely transformed into TiO_2_ after 5 h of H_2_O_2_ treatment.^22^ Further characterization employed a combination of structural, photoelectrical, and electrochemical techniques to elucidate the mechanisms behind the remarkable efficiency of our MXene-derived TiO_2_/Ti_3_CN heterostructure in the degradation of model organic pollutants. This research not only pioneers a strategy for fabricating a TiO_2_/Ti_3_CN heterostructure but also positions this material as a potential photocatalyst of paramount significance in the realm of environmental remediation.

## Experimental Section

### Synthesis of Ti_3_CN MXene

The detailed preparation of the Ti_3_AlCN MAX phase was reported elsewhere.^23^ We used a microwave-assisted hydrothermal technique to selectively etch out aluminum from the parental Ti_3_AlCN MAX phase. First, 400 mg of solid LiF was added slowly into 20 mL of HCl (6 M). After 10 min of stirring, we slowly added 1 g of Ti_3_AlCN powder (<45 μm), and the mixture was stirred for 10 min. After that, the mixture was sonicated in an ultrasonic bath for 5 min and transferred to the microwave reactor (Magnum II Microwave reactor, ERTEC, Poland). The microwave reactor was programmed at 150 °C, 480 W, and a reaction time of 4 h. After the etching process, the sedimented multilayered (ML) Ti_3_CN MXene flakes were washed several times with double distilled water (DDW) until the pH reached ∼6 and the remaining water was removed *via* freeze-drying, yielding ML Ti_3_CN MXene powders.

### Preparation of TiO_2_/Ti_3_CN Heterostructure

We performed the oxidation of ML-Ti_3_CN using H_2_O_2_ with different oxidation times. In detail, 100 mg of ML-Ti_3_CN was mixed with 1 mL of H_2_O_2_ (37% v/v) in 20 mL of DDW. The mixture was stirred for 3 min and bath-sonicated for 5 min. It was then loaded into the microwave reactor for hydrothermal treatment at 160 °C for 15 min (T-15M), 1 h (T-1H), and 5 h (T-5H). After the hydrothermal process, the obtained sediments were washed with DDW several times until the pH reached ∼6.

### Studies on the Morphology and Structure

The surface morphology of the samples and energy-dispersive X-ray (EDS) spectra were investigated by transmission electron microscopy (TEM, Tecnai G2, Eindhoven, The Netherlands) and scanning electron microscopy (SEM Hitachi S5500, Hitachi, Tokyo, Japan). The samples were mounted on a carbon-coated copper grid (Ted Pella Inc., Redding, CA). X-ray diffraction (XRD) analysis was carried out by a Bruker D8 Advanced diffractometer at Cu Kα - 0.154056 nm (Bruker, Billerica, MA). The ζ-potential and particle size distribution were measured with a Zetasizer (NANO ZS ZEN3500 analyzer, Malvern Instruments, Malvern, U.K.).

### Studies on the Chemical Composition

Bonding and vibrations in the samples were studied by using attenuated total reflectance-Fourier transform infrared (ATR-FTIR) spectroscopy with a diamond crystal (Nicolet iS5, Thermo Electron, Waltham, MA) and Raman spectroscopy (Renishaw InVia confocal Raman microspectrometer). The FTIR spectra were collected with a spatial resolution of 2 cm^–1^ in the 400–4000 cm^–1^ range; each spectrum reported is an average of 30 scans. Raman spectra were obtained with a 532 nm laser excitation using a 1200 mm^–1^ grating. Each spectrum was recorded with a 30 s acquisition time, 3 accumulations, and 1% laser power. Furthermore, the details of X-ray photoelectron spectroscopy (XPS) measurement are given in the Supporting Information (SI).

### Studies on Optical and Electrochemical Properties

The optical properties of the samples were studied with an ultraviolet–visible (UV–vis) (Evolution 220, Thermo Scientific) and photoluminescence (PL) spectrometer (F2700, Hitachi, Japan). The UV–vis data were taken in a diffuse reflectance (DRS) mode with an integrating sphere at a wavelength range of 220–1100 nm. PL measurements were carried out at room temperature in ambient conditions at a 380 nm excitation, and PL was recorded in the range of 400–700 nm. We optimized the PL measurement parameters to avoid the disturbance in the recorded spectra originating from light scattering of the light source.^24^ To study the charge-transfer dynamics and flat band potential, we performed electrochemical impedance spectroscopy (EIS) and Mott–Schottky (MS) measurements using an electrochemical workstation (VSP-300, Biologic, France). The details of the electrode preparation are listed in the SI.

### Photocatalytic Studies

For photocatalytic studies, 1 mL of model dye methylene blue (StanLab, Poland) (25 mg L^–1^) was mixed with 100 μL of colloidal Ti_3_CN or TiO_2_/Ti_3_CN catalyst in water (5 mg mL^–1^). The same parameter was used in the photocatalytic process with additional model dyes, such as methyl orange (Eurochem, Poland) and rhodamine B (Glentham Life Sciences, U.K.) (25 mg L^–1^). To begin the step toward commercial application, the commercial textile dye (Biel i Kolor, Steel System Marek Kacprzak, Poland) with a 10 times higher than model dyes was used (250 mg L^–1^). First, the adsorption equilibrium was established by stirring the mixture at 300 rpm for 15 min in the dark, followed by dye decomposition in the photochemical reactor (Photocube Photochemical reactor, ThalesNano, Hungary), equipped with eight light-emitting diodes (LEDs), each operating at an LED input power of 128 W. The photocatalytic experimental setup is presented in Figure S1. The photocatalysis was conducted in closed 4 mL borosilicate vials under simulated white light (400–700 nm, luminous flux of 1200 lm) and UV (365 nm, radiant flux of 44.8 W) irradiation with continued stirring (1350 rpm). Moreover, the reactor’s temperature was controlled at ∼30 °C. Notably, the borosilicate glass exhibits an absorption edge of ∼330 nm, shorter than the wavelength used in our photocatalytic study (Figure S2). This implies that the vial has negligible light absorption at the specific wavelength used in the photocatalytic study. The dye degradation was monitored by UV–vis absorption spectroscopy, taking the intensity of the characteristic dye peaks such as at 665 nm for methylene blue, 463 nm for methyl orange, 552 nm for rhodamine B, and 612 nm for commercial textile dye. Additionally, we performed similar experiments with multilayered Ti_3_C_2_, commercial P25 Degussa, and TiO_2_ anatase for comparison. To confirm the successful preparation of TiO_2_ anatase, we performed XRD measurement, and the diffractogram is presented in Figure S3. All of the photocatalytic process parameters were kept constant for each sample. The details of these referential sample preparations, reactive oxygen species analysis, and reusability studies are given in the SI.

## Results and Discussion

**Figure 1:**
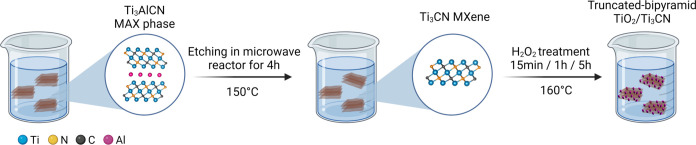
Schematic illustration of the synthesis process of a truncated-bipyramid TiO_2_/Ti_3_CN heterostructure derived from Ti_3_CN MXene. Created with BioRender.com.

**Figure 2:**
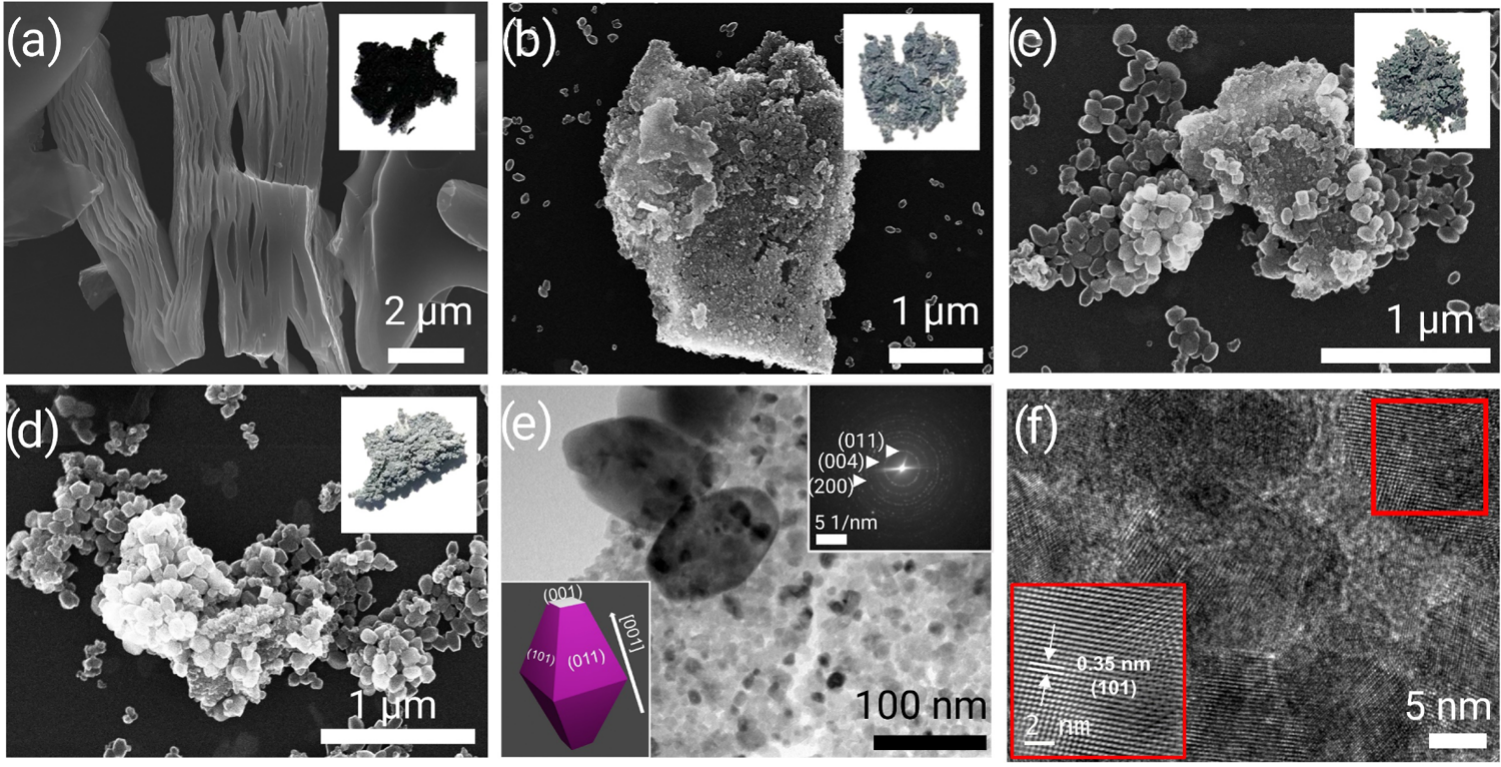
SEM image of (a) Ti_3_CN MXene, (b) T-15M, (c) T-1H, and (d) T-5H. (e) TEM image and (f) high-resolution TEM image of T-15M. The insets in panels (a–d) show powder digital optical photographs of Ti_3_CN_,_ T-15M, T-1H, and T-5H, respectively. The insets in panel (e) show the selective area electron diffraction (SAED) pattern of TiO_2_ anatase in T-15M and a schematic image of a truncated TiO_2_ bipyramid crystal. The inset in panel (f) shows the lattice fringes of TiO_2_ anatase nanocrystals.

The schematic illustration of the synthesis process of a bipyramidal TiO_2_/Ti_3_CN heterostructure derived from Ti_3_CN is presented in Figure 1. To confirm the successful Al etching of Ti_3_AlCN, we performed EDS measurements on Ti_3_CN. As expected, EDS elemental analysis reveals that the amount of Al is significantly reduced to a trace after HF etching (Figure S4). Moreover, the characteristic accordion-like structure of multilayered (ML) Ti_3_CN MXene is obtained after 4 h of microwave hydrothermal etching of Ti_3_AlCN, as shown in Figure 2a.

Next, we study the morphological changes of Ti_3_CN after performing a hydrothermal H_2_O_2_ oxidation treatment. After 15 min of oxidation (T-15M), the surface of Ti_3_CN begins to be decorated with truncated-bipyramid TiO_2_ nanocrystals (Figure 2b). The transformation of Ti_3_CN into TiO_2_ starts at atomic defects, acting as reactive sites for oxidation.^25^ In Ti_3_CN, the random distribution of C and N within the crystal lattice results in substitutional defects.^19^ These defects may account for the enhanced ability of Ti_3_CN to be *in* situ-decorated with TiO_2_ nanocrystals during hydrothermal oxidation treatment in just 15 min. Interestingly, T-15M still maintained a typical multilayered structure of MXene, forming the TiO_2_/Ti_3_CN heterostructure. As we increased the oxidation time to 1 h (T-1H), we observed an increasing amount of TiO_2_ and the formation of well-defined facets of TiO_2_ nanocrystals, as shown in Figure 2c. Further increasing the time to 5 h (T-5H), the Ti_3_CN surface was completely covered by truncated-bipyramidal TiO_2_ nanocrystals. This is also evidenced by charging artifacts in the SEM image (Figure 2d) and a significant increase in the oxygen content in the EDS spectra (Figure S5). The inset of Figure 2a–d presents the powders digital photographs of Ti_3_CN, T-15M, T-1H, and T-5H, respectively. As the transformation proceeded, we observed gradual color changes in the samples from black to light gray. The darker color stems from the unreacted Ti_3_CN MXene after the oxidation process. The same color shifting was also observed in the TiO_2_/Ti_3_C_2_ heterostructure obtained from *in situ* hydrolysis synthesis.^26^

We further carried out TEM measurements to study the atomic structure of TiO_2_ attached to the Ti_3_CN surface in detail. TEM image (Figure 2e) confirms the formation of truncated-bipyramid TiO_2_ anatase with well-defined facets, wherein (001) planes act as basal planes and (101) planes act as the edge planes. It is known that MXene synthesized by fluorine-based etchants is decorated by –F termination, which may lower the surface energy of highly energetical (001) facets.^27^ At the same time, H_2_O_2_ promotes the growth of nanocrystals along the [010] direction.^28^ The combination of these two factors results in the formation of the truncated-bipyramidal shape of TiO_2_ nanocrystals with high crystallinity, as observed in the high-resolution TEM image (Figure 2f).^28^ The selective area electron diffraction (SAED) pattern of TiO_2_ decorated on Ti_3_CN shows an anatase crystal structure (inset in Figure 2e). The interplanar spacing (*d*-spacing) of the TiO_2_ nanocrystal is 0.35 nm, corresponding to the (101) plane of TiO_2_ anatase.^29^ It is important to note that the high crystallinity of TiO_2_ nanocrystals is important for photocatalysis, as it can minimize the recombination rates of photogenerated electrons and holes.^30^

**Figure 3:**
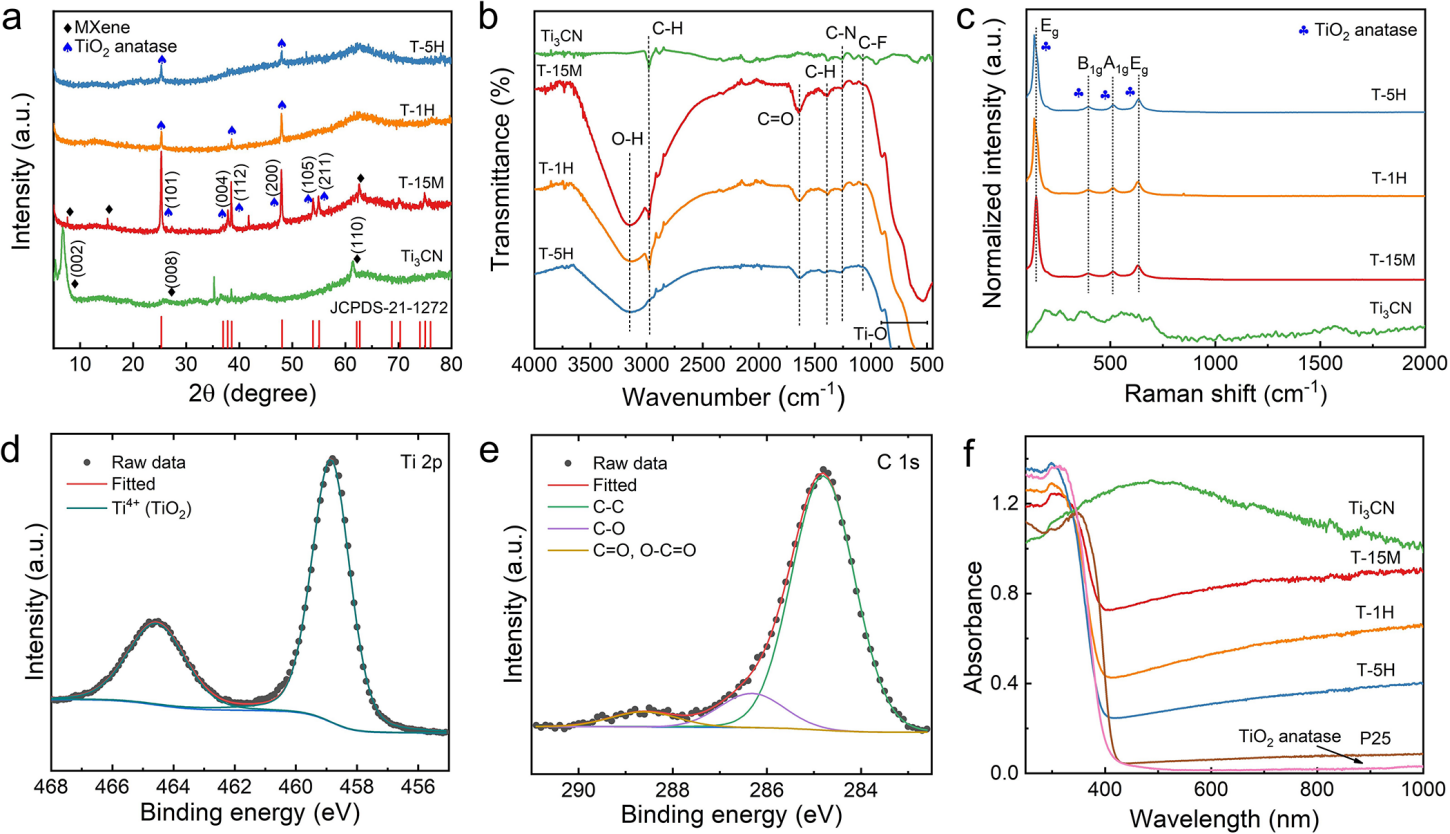
(a) XRD patterns, (b) ATR-FTIR, and (c) Raman spectra of Ti_3_CN, T-15M, T-1H, and T-5H. (d) Ti 2p and (e) C 1s XPS spectra of T-1H as a representative sample. (f) UV–vis absorption spectra of Ti_3_CN, T-15M, T-1H, and T-5H compared with the reference TiO_2_ anatase and P25.

The structure of Ti_3_CN MXene and its derived TiO_2_ was characterized using XRD (Figure 3a). First, we confirm the transformation of Ti_3_AlCN to Ti_3_CN. Ti_3_CN shows a strong (002) peak around a 2θ of 6.8°, shifted from 9.6° observed in Ti_3_AlCN (Figure S6). We do not observe the characteristic XRD peak of the Ti_3_AlCN phase at 39.5°, indicating the successful removal of the Al layers. After oxidizing Ti_3_CN, T-15M shows an anatase TiO_2_ pattern, matching with JCPDS-21–1272.^28^ Moreover, we observe a weak pattern of Ti_3_CN phases, indicating the generation of the TiO_2_/Ti_3_CN heterostructure after performing oxidation treatment.^27^ This result aligns with the SEM and TEM results, which indicate that Ti_3_CN still exists as a core or substrate in the heterostructure. In the case of T-1H and T-5H, the XRD peaks of Ti_3_CN MXene vanished due to the complete and uniform coverage of Ti_3_CN by TiO_2_ nanoparticles, as we previously discussed in the SEM analysis.

Next, we performed ATR-FTIR and Raman spectroscopy measurements to study the functional groups and chemical bonds in the material. Figure 3b shows the FTIR spectra of bare Ti_3_CN and oxidized Ti_3_CN samples. Ti_3_CN shows the presence of termination groups (–F and = O), as indicated by the C–F bond vibration (1078 cm^–1^) and C=O stretching (1640 cm^–1^), respectively.^31^ In contrast to the fresh Ti_3_CN sample, T-15M, T-1H, and T-5H exhibit Ti–O–Ti vibrations (600–1000 cm^–1^), indicating the formation of TiO_2_.^32^ Additionally, we observe a strong OH peak (surface hydroxyl or adsorbed water) in all of the TiO_2_/Ti_3_CN heterostructures. Surface OH groups are advantageous for promoting the hydrophilicity of TiO_2_ nanocrystals, thus ensuring enhanced photocatalytic activity.^33^ Interestingly, a similar oxidation treatment of the pristine Ti_3_AlCN MAX phase (before Al etching) does not lead to any noticeable changes in FTIR spectra (Figure S7). As expected, Ti_3_AlCN is stable upon microwave hydrothermal oxidation, and the digital photograph shows a dark-gray color of the MAX phase. Thus, we emphasize that transforming the Ti_3_AlCN MAX phase into Ti_3_CN MXene is a crucial step in obtaining well-crystalline TiO_2_ anatase *via* a microwave-assisted hydrothermal technique, as 2D Ti_3_CN MXenes are more reactive toward oxidizers and water compared to their bulk counterparts.^19,34^

Figure 3c shows the Raman spectra of the pristine Ti_3_CN and hydrothermally treated Ti_3_CN samples. The spectra reveal that only the TiO_2_ anatase phase was formed in T-15M, T-1H, and T-5H. In particular, the peaks at 148, 397, 513, and 633 cm^–1^ correspond to E_g_(1), B_1g_(1), A_1g_, and E_g_(3) modes of anatase TiO_2_, respectively.^35^ The absence of the characteristic peaks of rutile or brookite indicates the selectivity of microwave hydrothermal synthesis in producing TiO_2_ anatase from the MXene precursor. In addition, we did not observe any signs of typical D- and G- bands of solid graphitic carbon (Figure S8). The absence of these bands suggests that no solid graphitic carbon is present after microwave hydrothermal synthesis; instead, the formation of CH_4_ is more favored as previously investigated by Huang et al.^19^

XPS analysis was conducted to study the chemical states of the TiO_2_/Ti_3_CN heterostructure. XPS survey spectra (Figure S9) reveal the presence of Ti, O, C, N, and F. First, we examined the high-resolution Ti 2p XPS spectra of TiO_2_/Ti_3_CN (Figure 3d). Ti 2p spectra were fitted by a single doublet at 459.0 eV with a separation of 5.7 eV, indicating the Ti^4+^ oxidation state of TiO_2_.^36^ The typical Ti–C peak of MXene is not observed. This result is plausible, as XPS is a surface-sensitive technique, resulting in the measured signal originating from TiO_2_ nanocrystals, which completely cover the Ti_3_CN surface. Figure 3e shows the C 1s spectra of TiO_2_/Ti_3_CN. Herein, C 1s can be fitted by three components. The first peak at 285.0 eV indicates the presence of the C–C bond, whereas the peaks at 286.3 and 288.7 eV come from C–O and O–C=O or C=O bonds, respectively.^37^ These peaks come from oxidized carbon species adsorbed on the surface of TiO_2_/Ti_3_CN, which is typical for air-handled samples in the XPS measurement. The absence of the peak located around 290.7 eV suggests no graphitic carbon attached on the surface of the TiO_2_/Ti_3_CN heterostructure, in line with our Raman results.^38^

The O 1s XPS spectra can be fitted by three components (Figure S10a). The first peak centered at 530.0 eV indicates O–Ti, and the second peak at 531.5 eV indicates defective oxygen in TiO_2_ or O=C. The last peak located at 532.5 eV can be assigned to the surface hydroxyl group (O–H) or C–O-type bonds.^37,39,40^ Furthermore, we observed the presence of F^–^ ion in F 1s spectra, located at 684.4 eV (Figure S10b).^41^ The presence of F^–^ is attributed to the functionalization of Ti_3_CN following the Al etching of Ti_3_AlCN. N 1s can be fitted by two peaks located at 399.8 and 401.6 eV (Figure S10c). The former can be assigned to C–N bonds and the latter originated from NH_4_^+^.^37,39,41^ The presence of NH_4_^+^ as a degradation product of Ti_3_CN is in line with the result observed by Huang et al.^19^ However, the N 1s signal is considered very weak, and the analysis might be erroneous.

Next, we focused on the optical properties of Ti_3_CN MXene and hydrothermally treated Ti_3_CN samples compared to the reference samples, *i.e*., TiO_2_ anatase obtained by the sol–gel method and commercial P25. The optical absorption spectra (Figure 3f) reveal that in the solid state, Ti_3_CN shows strong absorption in the UV and is even stronger in visible light. We observe the absence of an absorption edge, indicating its metallic behavior as described in the previous theoretical paper.^42^ In contrast, an absorption edge is pronounced in T-15M, T-1H, and T-5H, which can be connected to the band-to-band transition in TiO_2_ anatase (∼400 nm).^43^

Furthermore, we observe that the visible absorption weakens by prolonging the hydrothermal oxidation treatment time. Interestingly though, even after 5 h of hydrothermal treatment, the visible absorption of T-5H is still stronger than that of P25 and reference TiO_2_ anatase. The weakening of visible absorption in TiO_2_/Ti_3_CN is attributed to the lowering amount of Ti_3_CN, which acts as a good photon absorber.^44,45^ In the UV region, P25 exhibits the weakest absorption. On the other hand, the TiO_2_/Ti_3_CN heterostructure shows a robust UV absorption intensity at wavelengths shorter than 400 nm. Therefore, based on the UV–vis results, we can conclude that TiO_2_/Ti_3_CN has a suitable absorption in the UV and visible spectrum ranges, stronger than benchmark P25 and TiO_2_ anatase.

We further estimated the optical band gap of the samples from the Tauc plot by assuming an indirect transition (Figure S11). To measure the band gap accurately, we applied baseline correction to all samples following the method proposed by Makula et al.^46^ T-15M shows an indirect optical band-gap value of 3.17 eV, lower than pure TiO_2_ anatase (3.20 eV). Here, the lower band gap of T-15M can be attributed to the interfacial effects between TiO_2_ and Ti_3_CN.^47^ By increasing the oxidation time, the band gap of the TiO_2_/Ti_3_CN heterostructure is blue-shifted to 3.22 eV (T-1H) and 3.23 eV (T-5H). This trend can be connected to more TiO_2_ generated on the Ti_3_CN surface.^48^ Photoluminescence (PL) spectra in Figure S12 show negligible signals for all samples. This finding is reasonable as MXene is a good conductor, and TiO_2_ is an indirect semiconductor with negligible luminescence at room temperature.^23,24^

The photocatalytic activity has been studied for other types of MXenes.^49^ In this study, the photocatalytic activity of TiO_2_/Ti_3_CN samples is evaluated based on the decomposition efficiency toward methylene blue (MB) as a model organic contaminant. First, we conducted MB adsorption studies in the dark (Figure S13). Ti_3_CN exhibits the highest dye adsorption compared with other samples. This can be attributed to the multilayered structure and negative ζ-potential of neat Ti_3_CN, which facilitate electrostatic interactions between MB and MXene.^50,51^ Conversely, we observe a decreasing trend in the adsorption of hydrothermally treated samples, where T-5H showed the weakest MB adsorption. This trend can be connected to the decoration of the MXene surface with TiO_2_ nanoparticles that may disrupt the layered structure of Ti_3_CN. Additionally, ζ-potential analysis indicates that the surface charge of TiO_2_/Ti_3_CN changed from negative to positive as the H_2_O_2_ treatment time was prolonged (Figure S14). Based on this knowledge, we started the photocatalytic processes after optimizing the adsorption/desorption equilibrium at 15 min.

**Figure 4:**
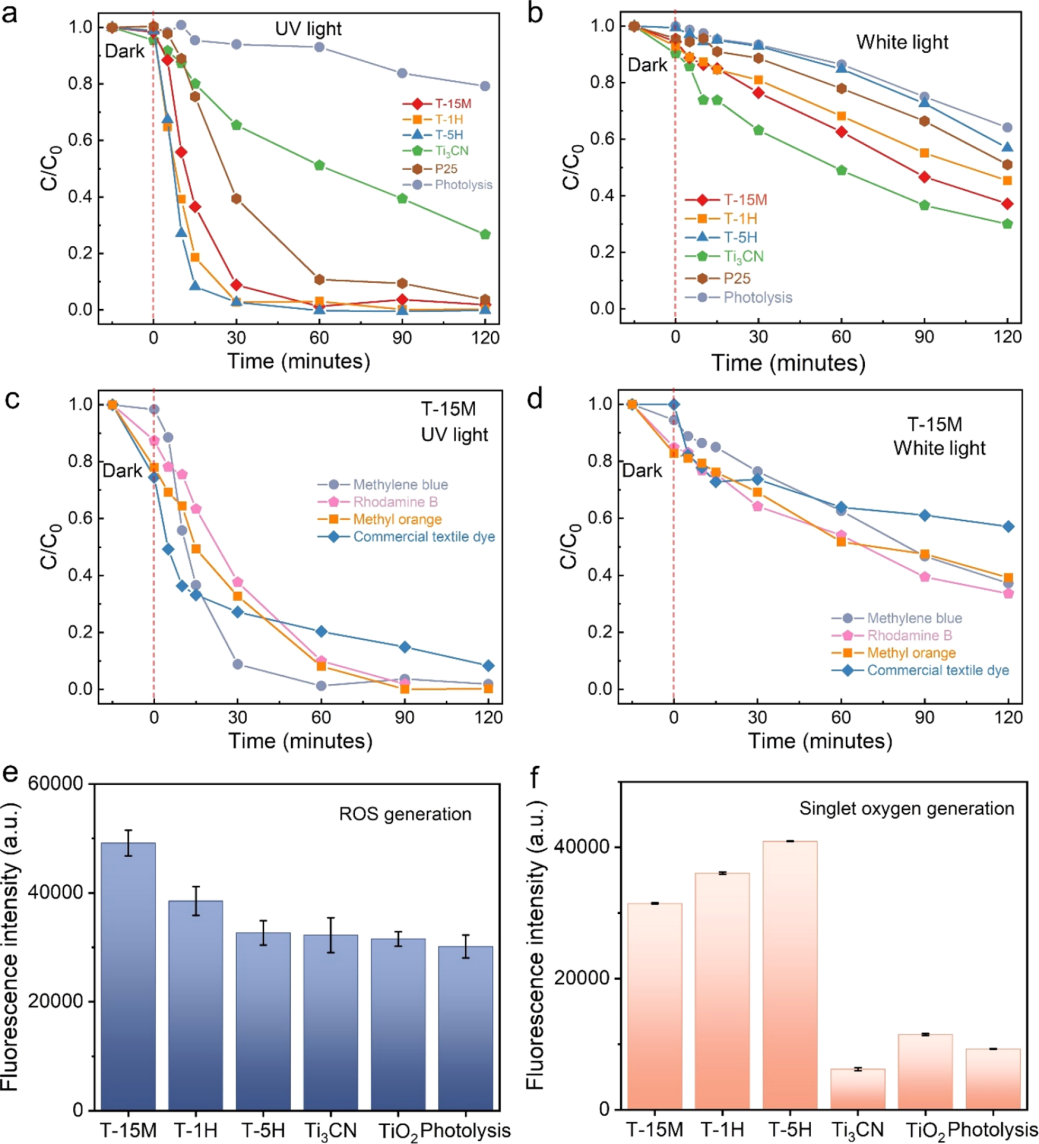
(a) Methylene blue decomposition in the presence of Ti_3_CN, T-15M, T-1H, T-5H, and reference P25 under (a) UV light and (b) simulated white light irradiation. Decomposition of various dyes, *i.e*., methylene blue, rhodamine B, commercial textile dye, and methyl orange, by T-15M under (c) UV light and (d) simulated white light irradiation. (e) Fluorescence intensity for (e) ROS and (f) singlet oxygen generation analysis.

Figure 4a shows MB decomposition under UV light. TiO_2_/Ti_3_CN effectively decomposed almost all MB within 30 min of irradiation. In particular, T-1H and T-5H decomposed 97% of MB, whereas T-15M shows a slightly lower MB decomposition efficiency of 91%. At the end of the photocatalysis process (after 2 h), all TiO_2_/Ti_3_CN samples achieved nearly ∼100% removal of MB. On the contrary, bare Ti_3_CN shows negligible MB decomposition despite demonstrating a high UV absorption based on the UV–vis results (Figure 3f). This observation is reasonable, considering that Ti_3_CN exhibits a metallic nature, and its observed negligible MB decomposition activity can be related to the superficial oxide on the Ti_3_CN surface. Reference samples (Ti_3_C_2_, P25, and TiO_2_ anatase; Figure S15) showed much lower decomposition performance. Although P25 eventually decomposed almost all dye (96% after 2 h of irradiation), its decomposition kinetic within the first 30 min is much slower than TiO_2_/Ti_3_CN. It is worth mentioning that P25 is one of the most efficient commercial photocatalysts and has been used frequently as a benchmark.^52^

The MB decomposition kinetics under UV light irradiation kinetics were fitted with a pseudo-first-order model (Figure S16a). The results show that TiO_2_/Ti_3_CN rapidly decomposed MB and reached a complete decomposition at 30 min of irradiation. T-5H exhibits the highest effective pseudo-first-order rate constant (*k*) of 0.125 min^–1^, followed by T-1H (0.122 min^–1^) and T-15M (0.084 min^–1^). Note that T-5H shows much higher *k* than reference samples, 21- and 4-fold higher than TiO_2_ anatase and P25, respectively. This observation is supported by the absorption spectra in which T-5H has the most substantial light absorption in the UV region. However, this factor does not solely determine the high MB decomposition efficiency of TiO_2_/Ti_3_CN, as the referential TiO_2_ anatase also displays a comparable UV absorption. Therefore, apart from robust UV absorption, we attribute the high photocatalytic activity of TiO_2_/Ti_3_CN to the following important factors: (1) high-crystallinity TiO_2_ anatase with well-defined facets, as observed in HR-TEM results. (2) Efficient photogenerated charge carrier transfer and separation owing to the presence of Ti_3_CN, as we will discuss in the photoelectrical and EIS analyses.

Under simulated white light irradiation (Figure 4b), Ti_3_CN exhibits the highest rate of decomposition of MB in solution, reaching 70% after 120 min, which can be attributed to the efficient dye adsorption by this sample (Figure S13). On the other hand, T-15M exhibits almost comparable photocatalytic activity with an efficiency of 63%. Interestingly, the photocatalytic performance deteriorates when we further increase the MXene oxidation treatment time: T-5H shows a photocatalytic performance that is almost similar to MB photolysis. This result is plausible, considering the band gap of T-5H (3.23 eV) located at the UV region. In addition, the performance of T-1H is comparable to the reference samples (TiO_2_ anatase and P25). These results indicate that besides the photocatalytic process, continuous adsorption and photolysis also play an essential part in the disappearance of MB in solution under simulated white light.^49^

The kinetics of MB decomposition under simulated white light is presented in Figure S16b, Supporting Information. Here, Ti_3_CN and T-15M exhibit a high *k* value compared to those of other samples, with values of 0.009 and 0.008 min^–1^, respectively. On the other hand, T-1H (0.006 min^–1^) and T-5H (0.004 min^–1^) show MB decomposition kinetics comparable to the reference TiO_2_ anatase (0.007 min^–1^) and P25 (0.005 min^–1^), indicating their limited efficiency under simulated white light. All of our *k* values are summarized in Table S1. We also compared our results with those of TiO_2_ photocatalysts and other state-of-the-art TiO_2_/MXene heterostructures, as presented in Table S2. Interestingly, our results reveal that the TiO_2_/Ti_3_CN heterostructure exhibits up to an ∼3.9-fold increase in photocatalytic performance in degrading MB under UV irradiation than P25, which is higher than any other TiO_2_/MXene heterostructures. After assessment of the photocatalytic performance of TiO_2_/Ti_3_CN, the reusability of the catalyst is investigated by performing subsequent cycles of the photocatalytic process. TiO_2_/Ti_3_CN shows comparable MB photodecomposition for the subsequent cycle of photocatalysis, indicating decent stability when subjected to the recycling process (Figure S17). This observation is in good agreement with previous studies that reported high environmental stability of bipyramid TiO_2_ nanocrystals.^33^

It is important to determine whether TiO_2_/Ti_3_CN could utilize white light to generate photoexcited charge carriers to understand the mechanisms of photocatalysis under white light. To elucidate the mechanism of action, we measured the current–voltage characteristics in the dark and under light irradiation (Figure S18). We choose T-15M for this study due to its high photocatalytic performance under visible and UV irradiation. Under UV irradiation, T-15M demonstrates an ∼2.5-fold higher photo-to-dark current ratio than bare indium tin oxide (ITO) substrate, indicating that T-15M is highly sensitive to UV light, as expected for partially degraded MXenes according to prior publications.^23,53^ Moreover, T-15M exhibited an ∼1.4-fold higher photo-to-dark current ratio than bare ITO under visible light irradiation. Therefore, electrical measurements indicate that UV and visible lights might induce photogenerated electrons and holes in T-15M. Notably, T-15M is more highly sensitive toward UV than white light, resulting in a lower MB decomposition performance under visible irradiation than UV. This is in line with optical analysis, which indicates the band-gap value of 3.17 eV for T-15M. Therefore, we conclude that the high photogenerated charge carrier production under UV light leads to the rapid degradation of MB by the TiO_2_/Ti_3_CN heterostructure.

We also conducted photocatalytic decomposition experiments on other synthetic dyes such as rhodamine B (RhB), a cationic dye, and methyl orange (MO), an anionic dye. Additionally, we evaluated the efficacy of TiO_2_/Ti_3_CN in decomposing commercial textile dyes with a concentration 10 times higher than those model dyes. Under UV irradiation (Figure 4c), TiO_2_/Ti_3_CN can fully decompose MB, RhB, and MO after 2 h of irradiation. Interestingly, a good result was also observed in decomposing commercial textile dye, where a decomposition rate reached 91.7%. Moving to white light irradiation (Figure 4d), TiO_2_/Ti_3_CN exhibited an almost identical performance in decomposing MB, RhB, and MO, while the commercial textile dye demonstrated lower decomposition compared to the other dyes.

Next, we analyzed the reactive oxygen species (ROS) responsible for dye degradation. ROS levels were quantified by using a commercial fluorescent probe. The principle behind this technique is that the fluorescence intensity emitted by the probe correlates with the concentration of ROS generated upon irradiation of the samples. Figure 4e indicates that the levels of ROS are higher in T-15M, T-1H, and T-5H compared to those in photolysis, Ti_3_CN, and TiO_2_. This suggests that more ROS are produced in T-15M, T-1H, and T-5H. During light irradiation, electrons are excited from the valence band to the conduction band of TiO_2_ and further react with O_2_ to produce superoxide radicals (O_2_^•–^). Singlet oxygen (^1^O_2_) is generated as one of the end products from superoxide radicals. Moreover, holes in the valence band react with adsorbed hydroxyl ions (OH^–^), generating hydroxyl radicals (^•^OH). These radicals can oxidize dissolved oxygen to produce oxygen-active species such as O_2_^•–^, ^1^O_2_, and ^•^OH, acting as crucial ROS for degrading pollutants.^54^

In order to detect the generation of ^1^O_2_, we employed a highly selective commercial fluorescent probe. T-15M, T-1H, and T-5H give a high fluorescence intensity compared with Ti_3_CN, TiO_2_, and photolysis, implying the significance of singlet oxygen formation (Figure 4f). Next, we analyzed the role of ^•^OH radical in photodecomposition mechanism of MB by adding 10 vol % isopropanol to the reaction solution.^55^ The ^•^OH radicals are well-known major active species in TiO_2_ photocatalysis in water that degrade organic molecules.^56^ The addition of isopropanol caused a significant reduction of photodecomposition rate under UV and simulated white light in the presence of T-15M, T-1H, and T-5H (Figure S19). Collectively, these results strongly suggest that reactive oxygen species, such as singlet oxygen and the ^•^OH radical, play a pivotal role in decomposing the dyes.

**Figure 5:**
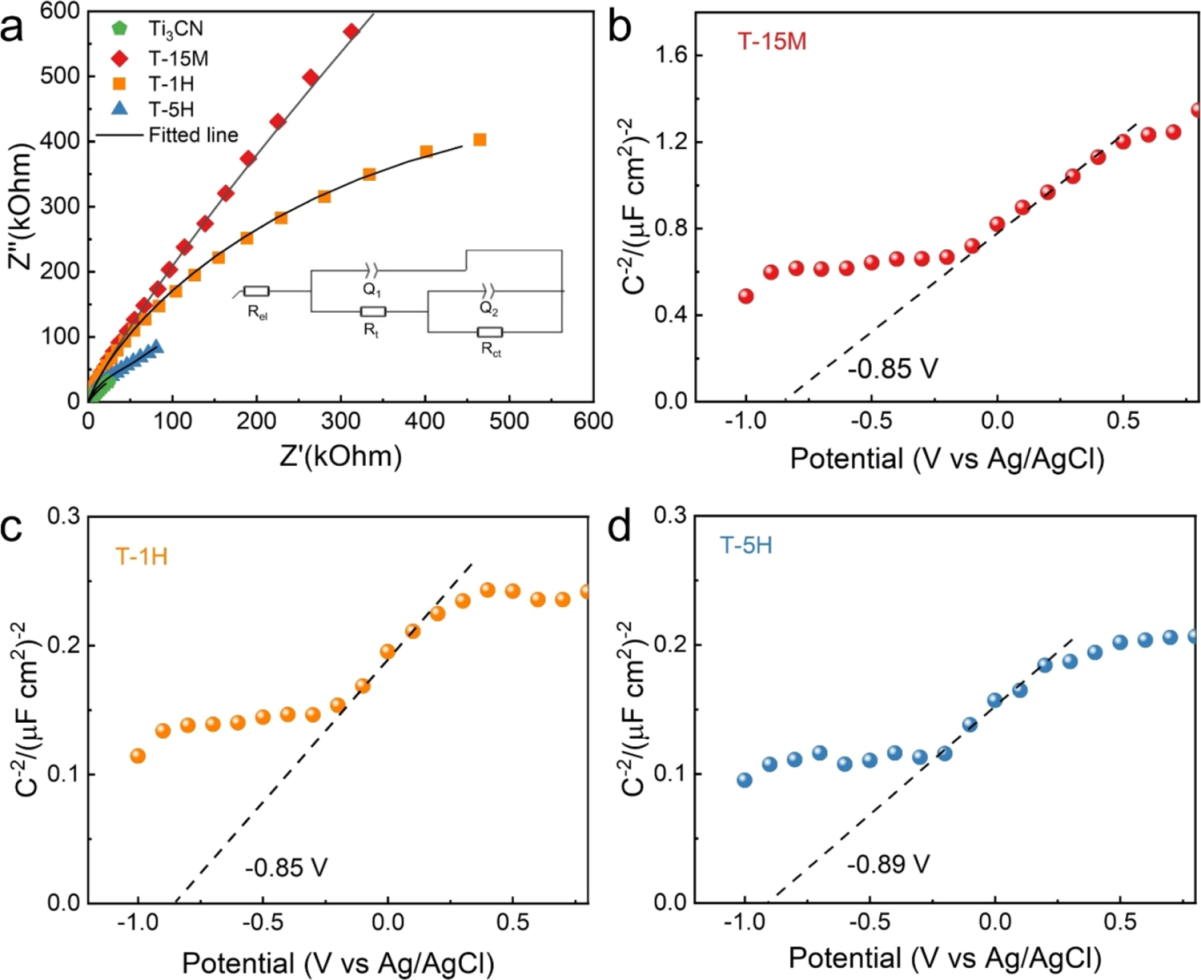
(a) Nyquist plot of the samples, with the inset showing an equivalent circuit for fitting analysis. The Mott–Schottky plot of (b) T-15M, (c) T-1H, and (d) T-5H. All of the Mott–Schottky measurements were carried out at a frequency of 1000 Hz.

After discussing the photocatalytic properties of TiO_2_/Ti_3_CN, we further explored the electrochemical behavior of the samples through electrochemical impedance spectroscopy (EIS). EIS measurements allow us to evaluate the photocatalysts’ charge-transfer resistance and interfacial properties. Figure 5a shows the Nyquist plots for Ti_3_CN, T-15M, T-1H, and T-5H. To analyze these spectra, we employed an embedded resistor–capacitor (RC) equivalent circuit, which allowed us to distinguish between bulk (*R*_t_) and charge-transfer resistance between the electrolyte and interface (*R*_ct_), as presented in the inset of Figure 5a. This model has been used to explain the presence of defect states or surface layers in metal oxide photocatalysts.^57−59^ Additionally, we used constant phase elements (*Q*_1_ and *Q*_2_) in the EIS fitting to account for the nonideal capacitive response of the materials.^58^

The obtained EIS fitting parameters are listed in Table S3. Ti_3_CN has the lowest *R*_t_ and *R*_ct_ values owing to its metallicity. However, despite its favorable electrical properties, Ti_3_CN lacks a band gap, rendering it unable to produce photogenerated charge carriers. Among the TiO_2_/Ti_3_CN samples, we observed the smallest arc radius in T-5H, followed by T-1H and T-15M, respectively. This result indicates that T-5H exhibits a higher efficiency in the charge-transfer process and reduced charge movement resistance.^60^ In detail, T-5H exhibits an *R*_ct_ of 186 kΩ, whereas T-1H and T-15M show much higher *R*_ct_ values of 1.5 and 26 MΩ, respectively. Therefore, we conclude that the rapid MB decomposition under UV light in T-5H is attributed to the superior charge-transfer process, high crystallinity, and robust UV absorption properties. The electron lifetime in the TiO_2_/Ti_3_CN samples is further studied by the Bode plot using the results of the EIS test (Figure S20). Here, the electron lifetime is inversely related to the frequency where the phase angle reaches maximum.^61^ Note that T-5H has a longer electron lifetime than the rest of the samples, indicating a lower recombination rate.

We further performed a Mott–Schottky analysis to reveal the electronic structure of both pristine Ti_3_CN and hydrothermally treated samples. The Mott–Schottky plot allowed us to determine the flat band potential (*V*_fb_) of the samples by extrapolating the linear portion of the plot to the *x*-axis.^62^ Obtained results show that all samples exhibit a positive slope in the Mott–Schottky plot, characteristic of n-type semiconductors.^63^ Specifically, T-15M (Figure 5b) and T-1H (Figure 5c) show similar *V*_fb_ of −0.85 V *vs* Ag/AgCl (equivalent to −0.65 V *vs* NHE at pH 7). Furthermore, T-5H exhibits a more negative *V*_fb_ value of −0.89 V *vs* Ag/AgCl (equal to −0.69 V *vs* NHE at pH 7) (Figure 5d). This more negative *V*_fb_ in T-5H implies a higher reduction potential of the photogenerated electron and increased reaction rate, potentially leading to improved photocatalytic activity compared to T-15M and T-1H.^12,64^ These results suggest that the oxidation treatment time may tune the surface charge and energy band structure, ultimately affecting the photocatalytic activity of the TiO_2_/Ti_3_CN heterostructures.

To further determine the band edge position, we assumed that the gap between the flat band potential (*V*_fb_) and the bottom of the conduction band (*E*_CB_) is considerably small.^62,65^ Furthermore, the valence band position (*E*_VB_) is calculated using the obtained band gap from UV–vis results by using the following empirical formula
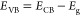
1

**Figure 6:**
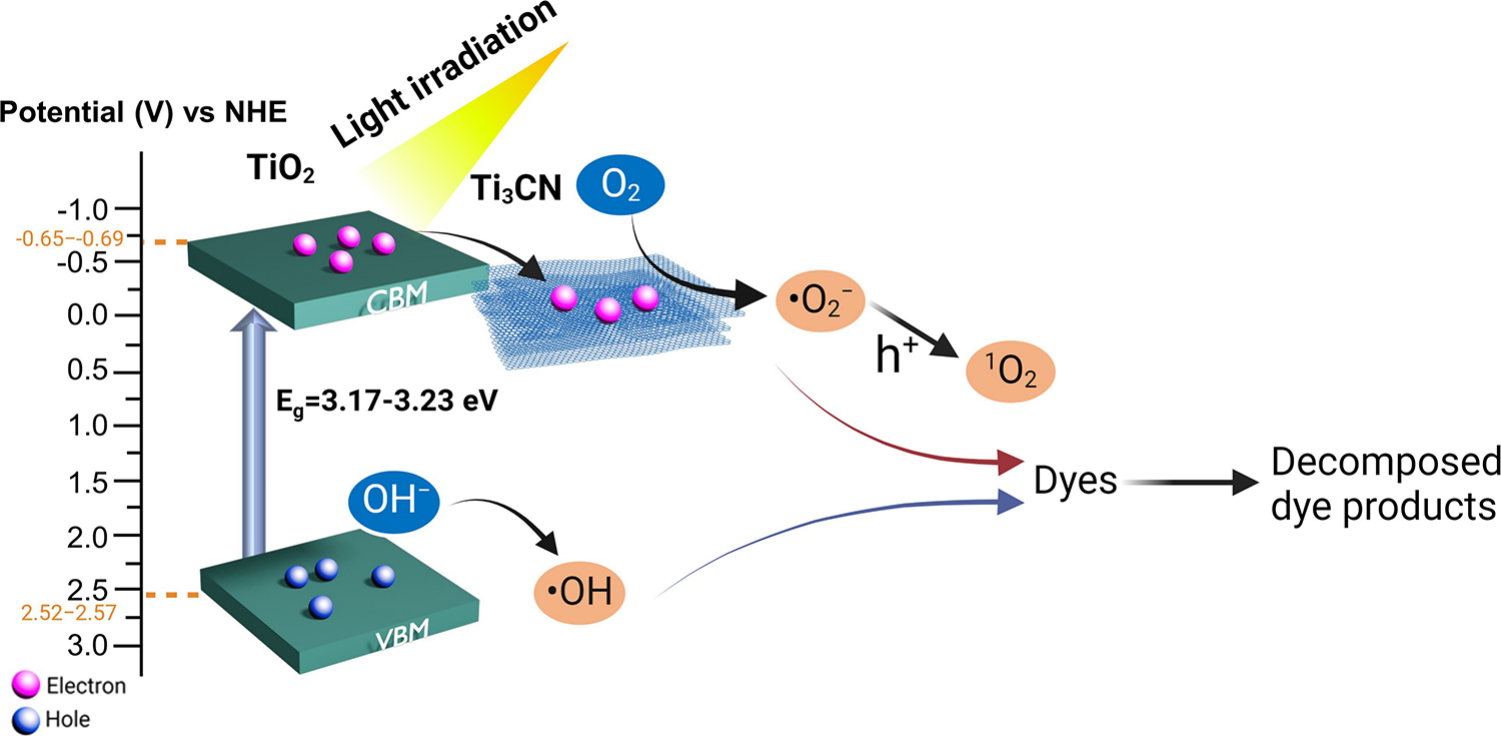
Proposed band structure schematic based on the Mott–Schottky and Tauc plots of the TiO_2_/Ti_3_CN heterostructure. Partly created with BioRender.com.

We obtained the *E*_VB_ values of T-15M, T-1H, and T-5H as 2.52, 2.57, and 2.54 eV, respectively. According to the band edge position of the samples, during the light irradiation, the excited electron in TiO_2_ can reduce O_2_ to produce ^•^O_2_^–^, due to the *E*_CB_ position being more negative than the potential of *E*_0_(O_2_/^•^O_2_^–^) (−0.33 V *vs* NHE).^66^ In addition, the photogenerated hole can oxidize the hydroxyl group owing to the more positive position of *E*_VB_ in T-15M, T-1H, and T-5H with respect to *E*_0_(^•^OH/H_2_O) (2.38 V *vs* NHE).^66^ Based on these results, we propose an energy diagram of TiO_2_/Ti_3_CN in Figure 6. During light irradiation, electron–hole pairs are generated in the conduction and valence bands of TiO_2_. The intimate interface between TiO_2_ and electrically conductive Ti_3_CN facilitated efficient electron transfer and formation of the Schottky barrier, enhancing the separation of photogenerated charges.^23,67^ The separated charge at TiO_2_ and Ti_3_CN is further used to produce ROS for synthetic dye degradation. The proposed photodegradation of synthetic dyes in the presence of TiO_2_/Ti_3_CN photocatalysts can occur through the following reactions^14,67,68^
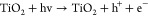
2
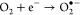
3
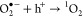
4
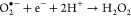
5
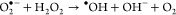
6
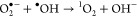
7
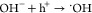
8
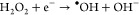
9
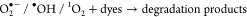
10It is important to note that we observed a trade-off between light absorption capacity and photogenerated charge carrier transfer efficiency in determining the photocatalytic activity of TiO_2_/Ti_3_CN. In this regard, T-15M shows good adsorption toward MB and high photodecomposition of MB under UV and simulated white lights. On the other hand, T-5H shows the fastest MB decomposition rate under UV light compared to those of other samples. Still, the performance of T-5H is limited by low adsorption capacity toward MB and low MB decomposition under simulated white light.

## Conclusions

In this study, we present a facile and controlled microwave-assisted hydrothermal method for the oxidative transformation of Ti_3_CN MXene into truncated-bipyramidal TiO_2_/Ti_3_CN heterostructures. The resulting TiO_2_ nanocrystals at the MXene surface exhibit a well-defined truncated-bipyramid anatase structure with exposed {001} and {101} facets. By varying the oxidation time, we could tailor the indirect optical band gap within the range of 3.17–3.23 eV while maintaining a constant treatment temperature of 160 °C. An extended oxidation time caused the flat band potential to shift to a lower negative energy level, resulting in a higher reduction potential for MB decomposition.

Electrochemical impedance spectroscopy provided insights into the charge-transfer process in TiO_2_/Ti_3_CN. The efficient charge separation of TiO_2_/Ti_3_CN enabled the high decomposition (97%) of methylene blue (MB) under just 30 min of UV light irradiation, which is 3.9-fold faster than P25. Moreover, under simulated white light conditions, these nanocrystals demonstrate an impressive efficiency of over 60% MB decomposition in just 2 h of irradiation, resulting in kinetics that are 1.5 times faster than the benchmark P25.

Altogether, the controlled microwave-assisted hydrothermal oxidation technique simplifies the *in situ* heterostructure formation and results in optically active nanocrystals with specific facets exposed. The significance of this advancement lies in its practical applications. The ability to tailor the optical properties of these nanocrystals by varying the oxidation time opens up opportunities for optimizing their performance under different environmental conditions. The shortened MB degradation times and higher efficiency under both UV light and simulated white light are promising, indicating that these nanocrystals could be used to create more efficient and sustainable remediation processes.

In summary, our study introduces an innovative strategy for harnessing the reactivity of MXenes to produce a highly crystalline, optically active, TiO_2_/Ti_3_CN heterostructure. This heterostructure serves as an efficient photocatalyst for the degradation of organic pollutants, offering great potential for enhancing environmental remediation processes. Our research contributes to the development of advanced photocatalytic materials and demonstrates the power of innovative materials science in addressing pressing environmental challenges.

## Supporting Information Available

Experimental setup for the photocatalytic decomposition of methylene blue; absorbance of borosilicate glass vial used for the photocatalytic study; XRD spectra of referential TiO_2_ anatase; EDS spectra of Ti_3_AlCN, Ti_3_CN, and TiO_2_/Ti_3_CN; FTIR spectra of Ti_3_AlCN treated with H_2_O_2_ oxidation treatment; magnified view of Raman spectra of TiO_2_/Ti_3_CN; XPS survey spectra; O 1s, F 1s, and N 1s of TiO_2_/Ti_3_CN; Tauc plot and PL spectra of TiO_2_/Ti_3_CN; adsorption of MB on photocatalysts; ζ-potential of Ti_3_CN and TiO_2_/Ti_3_CN; photocatalytic results of referential samples; reusability of TiO_2_/Ti_3_CN for subsequent photocatalytic cycles; photoelectric results of TiO_2_/Ti_3_CN; Bode plots of TiO_2_/Ti_3_CN; experimental setup for photocatalytic studies; rate constant (*k*) of MB decomposition in the presence of photocatalysts under UV and simulated white light irradiation; comparison of photocatalytic performance; and EIS fitting parameters of Ti_3_CN and TiO_2_/Ti_3_CN heterostructures (PDF)
