# Supplementary material for: Microwave-Assisted Hydrothermal Synthesis of Photocatalytic Truncated-Bipyramidal TiO2/Ti3CN Heterostructures Derived from Ti3CN MXene
Source: Langmuir. 2024 Oct 4;40(41):21547–58. doi: 10.1021/acs.langmuir.4c02444 (PMC11483757; doi:10.1021/acs.langmuir.4c02444)
Supplement: Supplementary file 1 — la4c02444_si_001.pdf [file la4c02444_si_001.pdf]

Supporting Information

Microwave-Assisted Hydrothermal Synthesis of Photocatalytic Truncated Bipyramidal TiO_2/Ti_3CN Heterostructures Derived from Ti_3CN MXene

Muhammad Abiyyu Kenichi Purbayanto[1,2*], Madhurya Chandel[1,2], Dominika Bury[1,2], Anna Wójcik[3], Dorota Moszczyńska[1], Anika Tabassum[4], Vadym N. Mochalin[5,6], Michael Naguib[4], Agnieszka Maria Jastrzębska[1,2*]

[1] Faculty of Materials Science and Engineering, Warsaw University of Technology, Wołoska 141, Warsaw, 02-507, Poland

[2] Faculty of Mechatronics, Warsaw University of Technology, św. Andrzeja Boboli 8, 02-525 Warsaw, Poland

[3] Polish Academy of Sciences, Institute of Metallurgy and Materials Science, W. Reymonta 25, 30-059 Cracow, Poland

[4] Department of Physics and Engineering Physics, Tulane University, New Orleans, LA 70118, USA

[5] Department of Chemistry, Missouri University of Science and Technology, Rolla, MO, 65409 USA

[6] Department of Materials Science and Engineering, Missouri University of Science and Technology, Rolla, MO, 65409 USA

*email: muhammad_abiyyu.kenichi.dokt@pw.edu.pl, agnieszka.jastrzebska@pw.edu.pl

List of Supporting Figures and Tables

Supporting Figures

Figure S1. Experimental set-up for photocatalytic decomposition of methylene blue.

Figure S2. Absorbance of borosilicate glass vial used for photocatalytic study.

Figure S3. XRD spectra of referential TiO_2 anatase sample obtained by the sol-gel method.

Figure S4. EDS spectra of Ti_3CN measured on carbon-coated copper grid. Excessive C contributions come from carbon coated copper grid.

Figure S5. EDS spectra of (a) T-15M, (b) T-1H, and (c) T-5H derived from Ti_3CN measured on carbon-coated copper grid. Observable Cu and excessive C contributions come from carbon coated copper grid.

Figure S6. XRD patterns of Ti_3AlCN and Ti_3CN. The red dots denote the diffraction peaks from TiN or TiC.

Figure S7. FTIR spectra of Ti_3AlCN and Ti_3AlCN after H_2O_2 treatment for 15 minutes. The inset shows the digital photograph of the sample after H_2O_2 treatment.

Figure S8. Raman spectra of T-15M, T-1H, and T-5H, indicating the absence of D- and G-bands of carbon materials.

Figure S9. XPS survey spectra of TiO_2/Ti_3CN heterostructure.

Figure S10. (a) O 1s, (b) F 1s, and (c) N 1s XPS spectra of TiO_2/Ti_3CN heterostructure.

Figure S11. Tauc plot of Ti_3CN, T-15M, T-1H, and T-5H compared with the reference TiO_2 anatase (sol-gel) and P25. The Tauc plot of Ti_3CN is missing due to the absence of the absorption edge

Figure S12. PL spectra of Ti_3CN, T-15M, T-1H, and T-5H compared with the reference TiO_2 anatase (sol-gel) and P25.

Figure S13. Adsorption of methylene blue on the sample in the dark condition.

Figure S14. Zeta potential of Ti_3CN, T-15M, T-1H, and T-5H.

Figure S15. (a) Decomposition and (b) kinetic of the methylene blue decomposition in the presence of T-15M, Ti_3C_2 and TiO_2 anatase under UV light. (c) decomposition and (d) kinetic under simulated white light irradiation. The decomposition without the catalysts (photolysis) is also plotted in the graph.

Figure S16. (a) Kinetic of methylene blue decomposition under (a) UV light and (b) simulated white light irradiation in the presence of Ti_3CN, T-15M, T-1H, T-5H, and P25. The decomposition without the catalysts (photolysis) is also plotted in the graph.

Figure S17. Reusability of T-15M in five subsequent photocatalytic cycles towards methylene blue.

Figure S18. The current-voltage curve of bare ITO and T-15M under (a) visible and (b) UV light irradiation. Photo-to-dark current ratio of bare ITO and T-15M under (c) visible and (d) UV light irradiation.

Figure S19. Photocatalytic MB decomposition with the presence of isopropanol as •OH scavenger under (a) UV and (b) simulated white light.

Figure S20. Bode EIS plots of T-15M, T-1H, and T-5H.

Supporting Tables

Table S1. Rate constant of MB decomposition in the presence of photocatalysts under UV and simulated white light irradiation.

Table S2. Comparison of photocatalytic performance for dye degradation between our work and previously reported TiO_2-based photocatalysts.

Table S3. EIS fitting parameters of Ti_3CN, T-15M, T-1H, and T-5H.

Experimental procedures

A. Preparation of referential samples

***Preparation of multilayered Ti_3C_2* MXene**

Ti_3AlC_2 MAX phase (<45μm) was added to concentrated hydrofluoric acid 48% v/v (Sigma-Aldrich). The etching process was performed for 24 h under stirring of 250 RPM. After finishing the etching process, the sediment was washed multiple times with double-distilled water (DDW) until the pH reached ~6.

Preparation of TiO_2 anatase nanoparticles

TiO_2 nanoparticle was prepared by using the sol-gel technique. In particular, Titanium (IV) isopropoxide (TTIP) (97%, Sigma-Aldrich) and DDW were used as a titanium precursor and hydrolysis medium.[1] First, 5 mL of TTIP was added to 100 mL of isopropanol under stirring of 300 RPM for 1 h. Then, 400 mL of DDW was added quickly to the mixture and stirred (300 RPM) for the next 30 minutes. The mixture was then aged for 3 h under an ambient condition. The precipitates were washed with DDW and further dried at 60 °C overnight. The obtained powders were crushed using a mortar and pestle. In order to initiate the crystallization, the powders were calcinated in a muffle furnace at 450 °C for 2 h with a heating rate of 5 °C/min in an air atmosphere.

B. Additional characterization methods

XPS surface analysis

The XPS analyses were carried out in a PHI VersaProbeII Scanning XPS system using monochromatic Al Kα (1486.6 eV) X-rays focused to a 100 μm spot. The photoelectron take-off angle was 45° and the pass energy in the analyzer was set to 117.50 eV (0.5 eV step) for survey scans and 46.95 eV (0.1 eV step) to obtain high energy resolution spectra for the C 1s, O 1s, Ti 2p, Cl 2p, F 1s, and N 1s. A dual beam charge compensation with 7 eV Ar^+ ions and 1 eV electrons was used to maintain a constant sample surface potential regardless of the sample conductivity. All XPS spectra were charge referenced to the saturated aliphatic carbon C 1s line

at 285.0 eV. The operating pressure in the analytical chamber was less than $4.5x10^{-9}$ mbar. Deconvolution of spectra was carried out using PHI MultiPak software (v.9.9.3). Spectrum background was subtracted using the Shirley method. Before the analysis and introduction to the UHV XPS chamber, the samples were drop-casted onto the silicon wafer and dried on a hot plate. XPS analysis was conducted without performing prior argon sputtering on the sample. The information depth of the XPS analysis, within the geometry of spectrometer can be estimated at about 5 nm.

Preparation of the samples for electrochemical study

The prepared samples were coated on glassy carbon (GC) as a working electrode, while Ag/AgCl (saturated in KCl) and a platinum wire were used as reference and counter electrodes, respectively. Here, 0.1 M KCl was used as a supporting electrolyte. To prepare the working electrode, the samples were sonicated for 30 minutes. Then 10 μL of the solution was drop-casted on the GC and dried overnight at the ambient temperature. EIS measurement was carried out at open circuit potential with 10 mV amplitude and the frequency of 100 kHz to 0.05 Hz. Moreover, Mott-Schottky measurement analysis was done under the frequency of 1000 Hz. All the measurements were done in the dark at room temperature.

Determination of methylene blue decomposition kinetic rate

The kinetic plots were obtained by utilizing a pseudo-first-order kinetic model.[2]

$$ln\left(\frac{C}{C_0}\right) = -kt \quad \text{(S1)}$$

where C and C_0 denote the initial dye concentration and concentration at a particular time (*t*).

Electrical measurement

First, 1 × 1 cm^2 indium tin oxide (ITO) substrates (3D nano, Poland) were cleaned by ultrasonication in acetone, isopropanol, and double distilled water for 5 minutes each. Then, the substrates were activated by UV/O_3 treatment for 1 h. The distance between the substrate and

the UV lamp was 2.5 cm. TiO_2/Ti_3CN powder was uniformly dispersed in ethanol with a concentration of 5 mg mL^{-1} and treated with an ultrasonic bath for 30 minutes. 40 μL of the dispersion was drop-casted by micropipette, while the substrate was heated at 80 °C to ensure layer uniformity.[3] The drop-casting process was repeated seven times, and the sample was dried at 80°C in an ambient atmosphere overnight. The current-voltage curve was measured by Keithley SMU 2450 under dark or light irradiation. In particular, the sample was irradiated by simulated white light (400 – 700 nm, luminous flux of 1200 lumens) and UV light (365 nm, radiant flux of 44.8 W), similar to photocatalytic experiments.

Reactive oxygen species, singlet oxygen analysis and reusability studies

The presence of reactive oxygen species (ROS) was measured with CellROX Green Reagent test (Thermo Fisher Scientific, MA, USA) with 95-100% dimethyl sulfoxide (C_2H_6OS, DMSO). The analysis was conducted after 45 seconds of photocatalytic process with DI under UV irradiation. The short time of the irradiation caused the generation of the largest amount of ROS at the initiation of the photocatalysis process.[4] After photocatalytic process, 200 μL of samples and 2 μL of fluorescence probe were transferred to each well of a multiwall plate. Each plate was subsequently placed in darkness for 30 minutes to facilitate a colorimetric reaction involving hydroxyl radicals. The ROS were checked utilizing an Infinite 200 PRO multiplate reader (Tecan, Männedorf, Switzerland), chosen for its bromocresol indicator's vivid green photostable fluorescence characteristics. The measurement parameters included an excitation wavelength of 485 nm, emission wavelength of 520 nm, excitation bandwidth of 20 nm, emission bandwidth of 20 nm, integration time, temperature set at 26.9 °C, shaking 10 seconds, and an amplitude of 4 mm. Results are presented as the mean FL intensity ± standard deviation derived from three replicates.

The singlet oxygen level was confirmed by a fluorescence method using Singlet Oxygen Sensor Green fluorescence reagent (Thermo Fisher Scientific, MA, USA). The as-received reagent is

dissolved in methanol (99.8% pure, POCH, Poland) to make a stock solution of ~5 mM. Then, the 200 μL of samples after 45 sec. of UV irradiation with 1 mL of water and 2 μL of fluorescence reagent was added to each well of a multiwall plate and then left in the dark for 60 minutes. Finally, the measurements were prepared at the same parameters as for singlet oxygen analysis.

The influence of •OH radical on photocatalytic process was studied by adding 10% (v/v) isopropanol to the reaction solution.[5] Furthermore, to perform the reusability studies, the spent catalysts were washed several times with ethanol and DDW and collected by centrifugation (6000 RPM) before their next use.

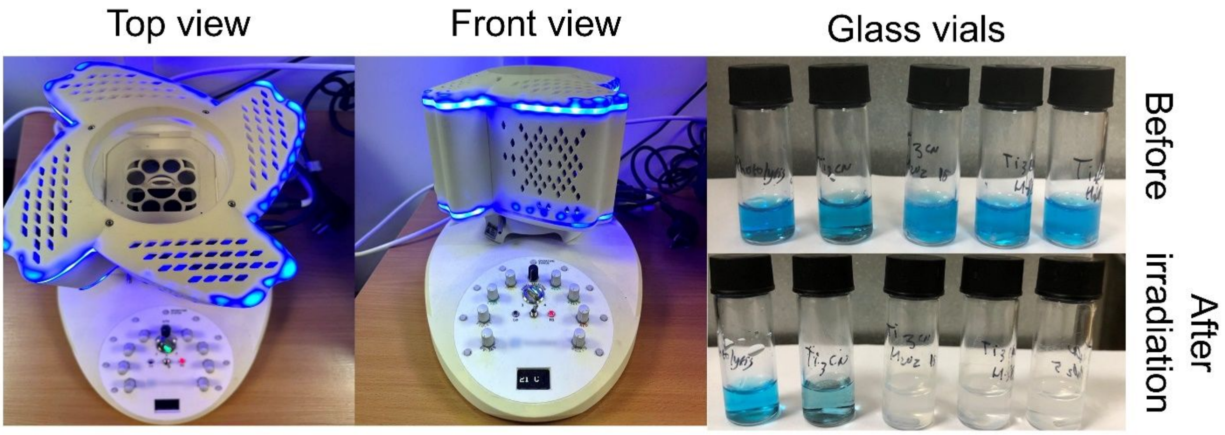

Figure S1. Experimental set-up for photocatalytic decomposition of methylene blue.

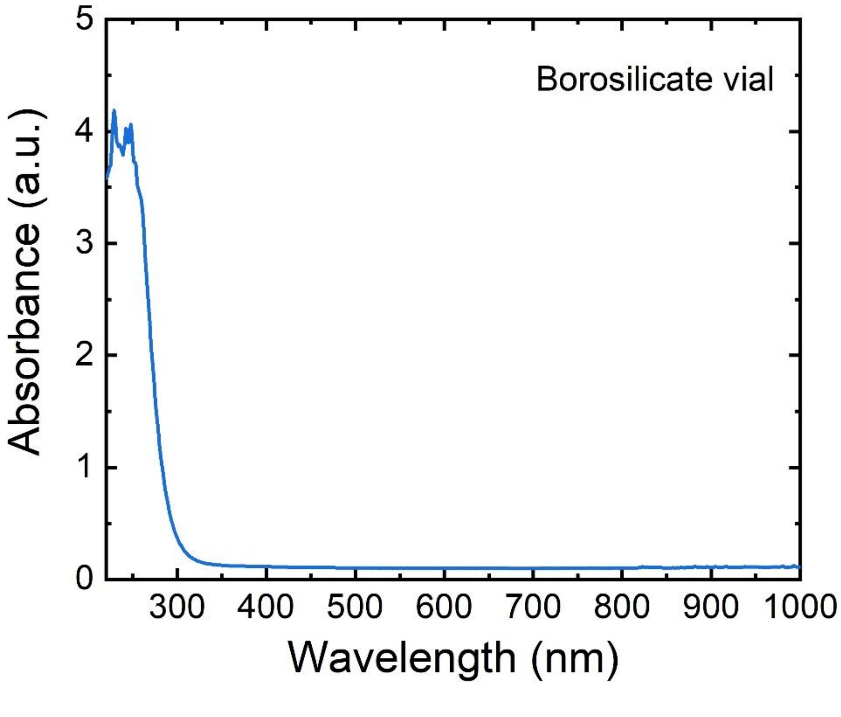

Figure S2. Absorbance of borosilicate glass vial used for photocatalytic study.

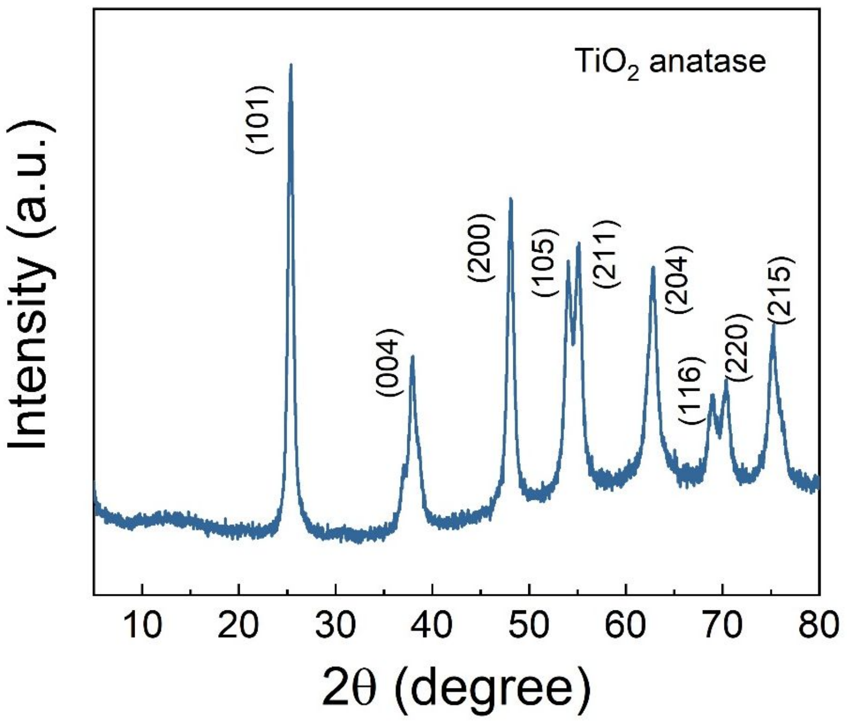

Figure S3. XRD spectra of referential TiO_2 anatase sample obtained by the sol-gel method.

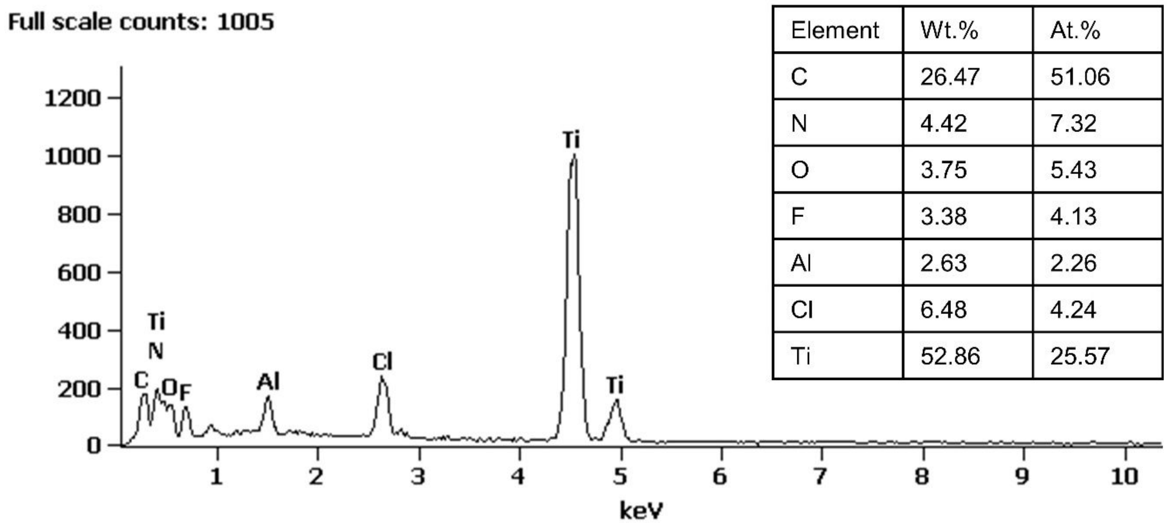

Element	Wt.%	At.%
C	26.47	51.06
N	4.42	7.32
O	3.75	5.43
F	3.38	4.13
Al	2.63	2.26
Cl	6.48	4.24
Ti	52.86	25.57

Figure S4. EDS spectra of Ti_3CN measured on carbon-coated copper grid. Excessive C contributions come from carbon coated copper grid.

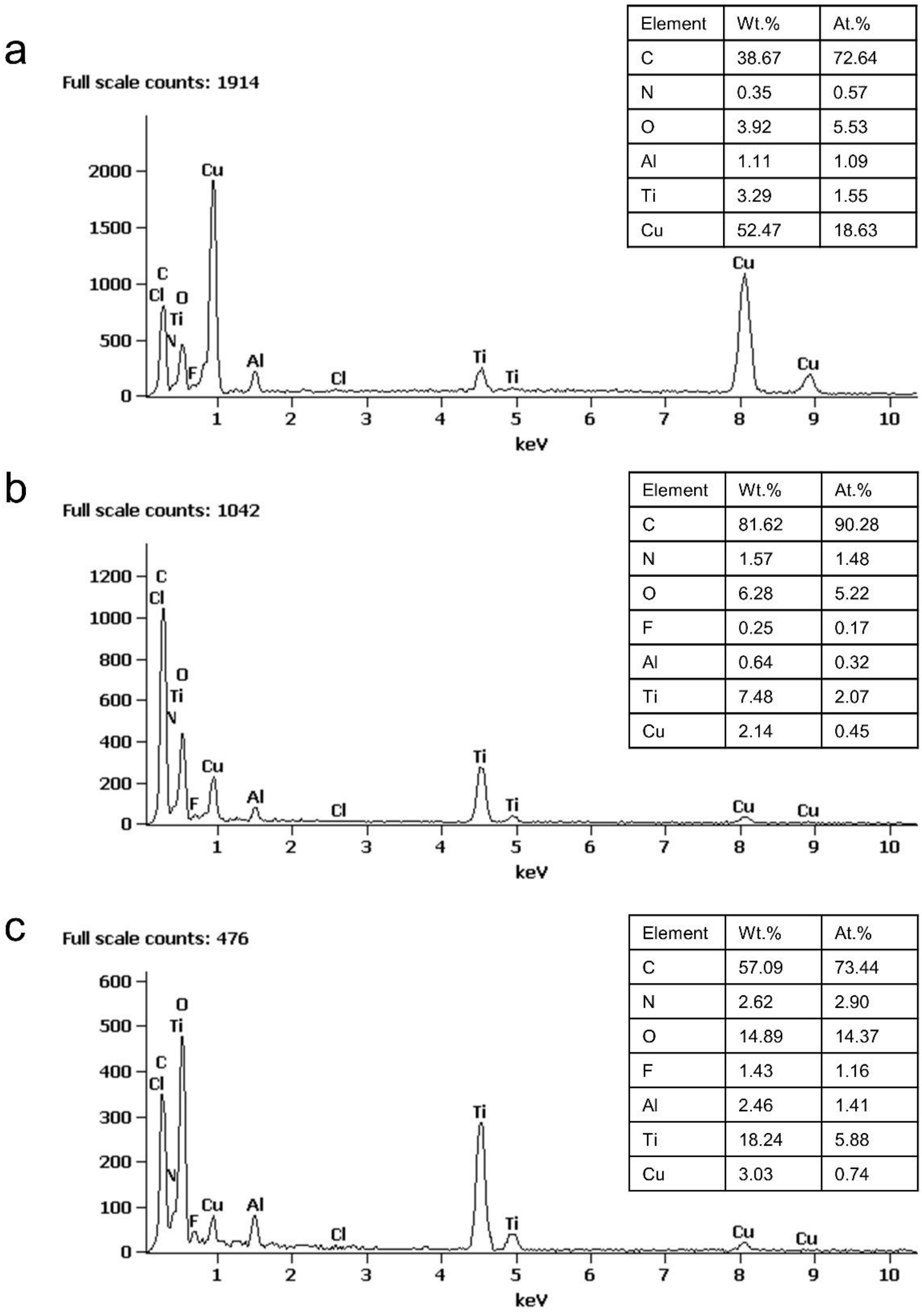

a

Element	Wt.%	At.%
C	38.67	72.64
N	0.35	0.57
O	3.92	5.53
Al	1.11	1.09
Ti	3.29	1.55
Cu	52.47	18.63

b

Element	Wt.%	At.%
C	81.62	90.28
N	1.57	1.48
O	6.28	5.22
F	0.25	0.17
Al	0.64	0.32
Ti	7.48	2.07
Cu	2.14	0.45

c

Element	Wt.%	At.%
C	57.09	73.44
N	2.62	2.90
O	14.89	14.37
F	1.43	1.16
Al	2.46	1.41
Ti	18.24	5.88
Cu	3.03	0.74

Figure S5. EDS spectra of (a) T-15M, (b) T-1H, and (c) T-5H derived from Ti_3CN measured on carbon-coated copper grid. Observable Cu and excessive C contributions come from carbon coated copper grid.

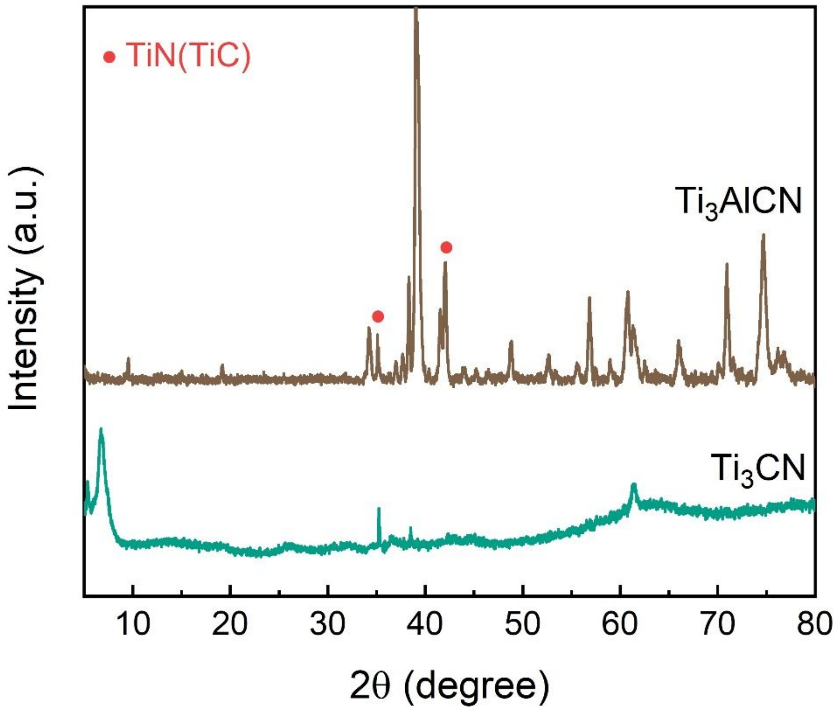

Figure S6. XRD patterns of Ti_3AlCN and Ti_3CN. The red dots denote the diffraction peaks from TiN or TiC.

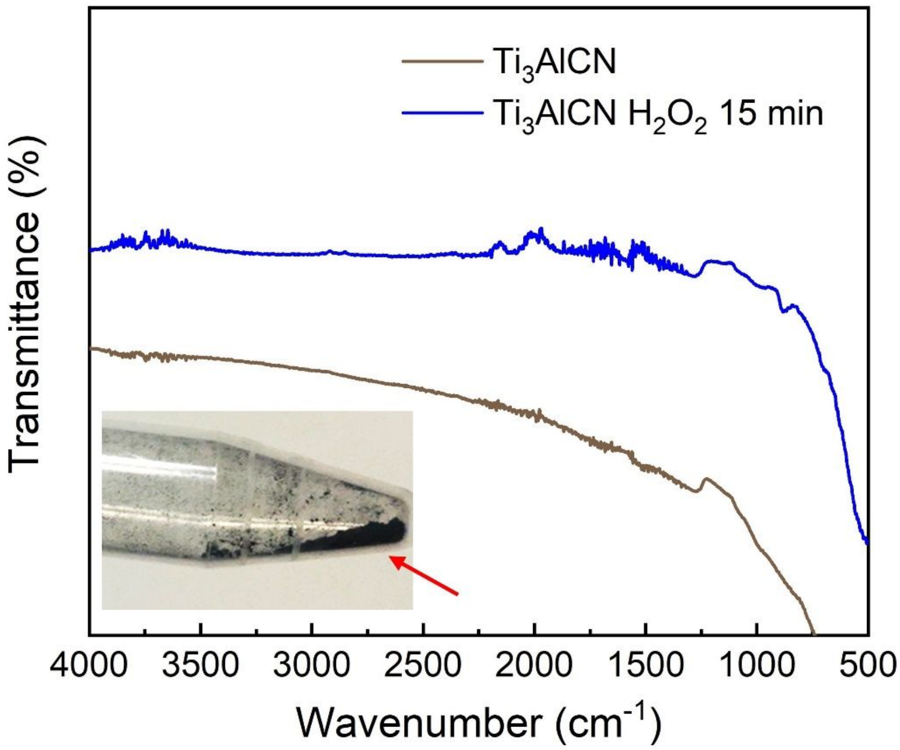

Figure S7. FTIR spectra of Ti_3AlCN and Ti_3AlCN after H_2O_2 treatment for 15 minutes. The inset shows the digital photograph of the sample after H_2O_2 treatment.

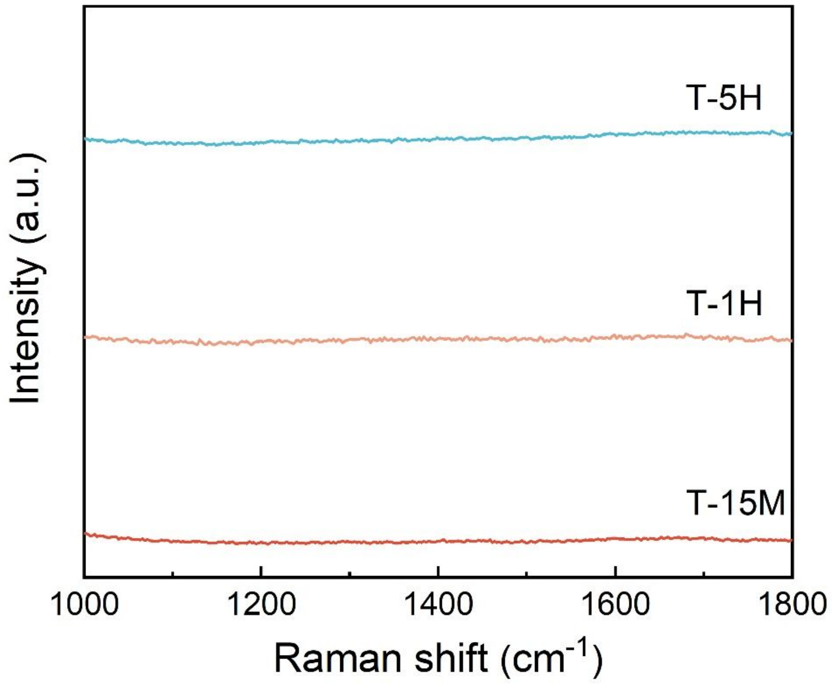

Figure S8. Raman spectra of T-15M, T-1H, and T-5H, indicating the absence of D- and G-bands of carbon materials.

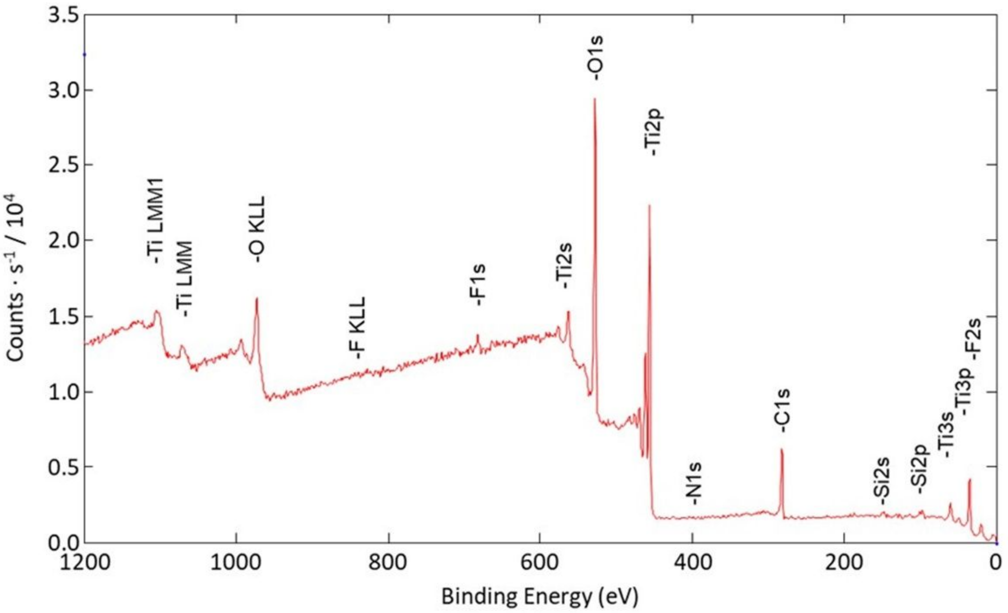

Figure S9. XPS survey spectra of TiO_2/Ti_3CN heterostructure.

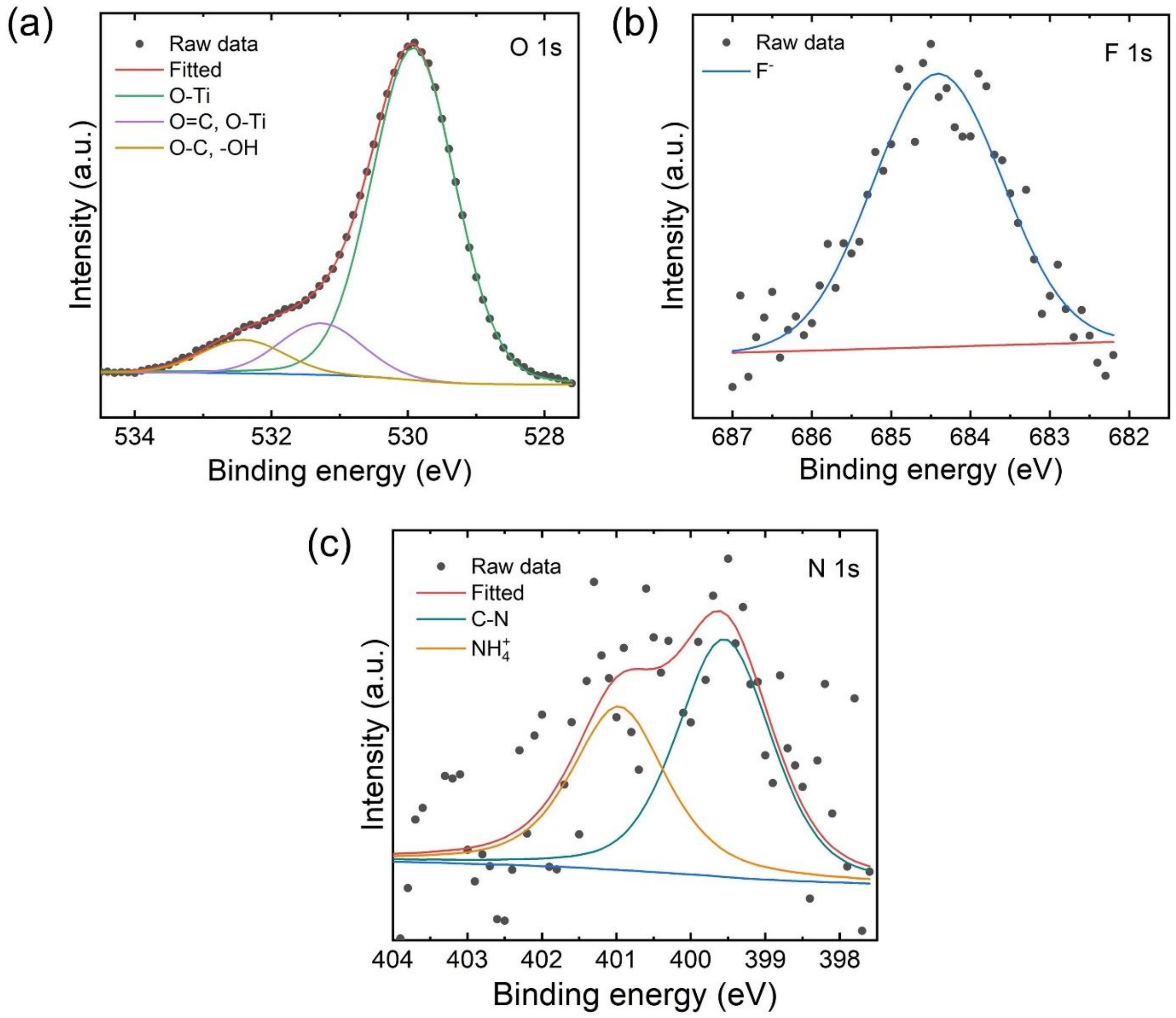

Figure S10. (a) O 1s, (b) F 1s, and (c) N 1s XPS spectra of TiO_2/Ti_3CN heterostructure.

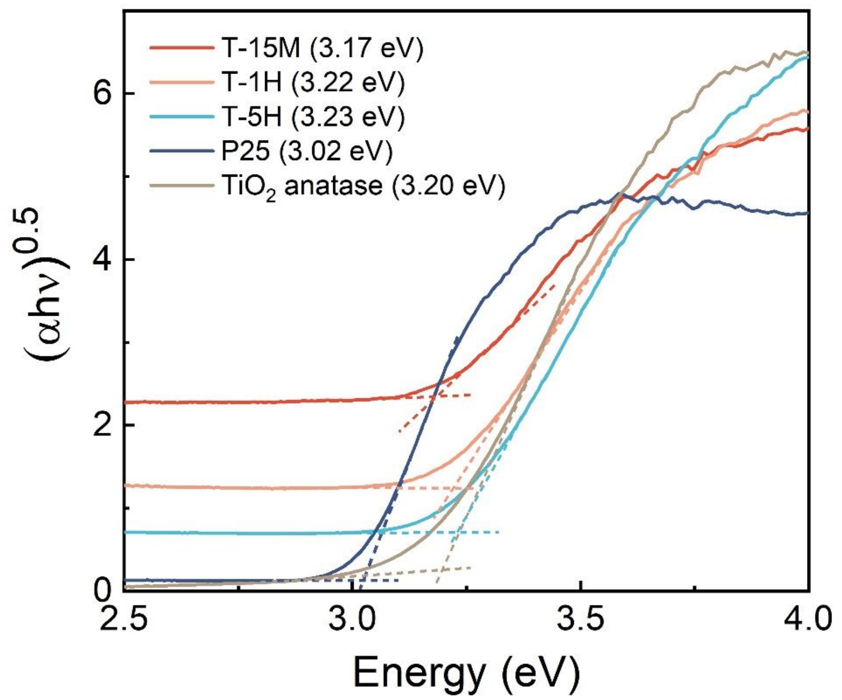

Figure S11. Tauc plot of Ti_3CN, T-15M, T-1H, and T-5H compared with the reference TiO_2 anatase (sol-gel) and P25. The Tauc plot of Ti_3CN is missing due to the absence of the absorption edge

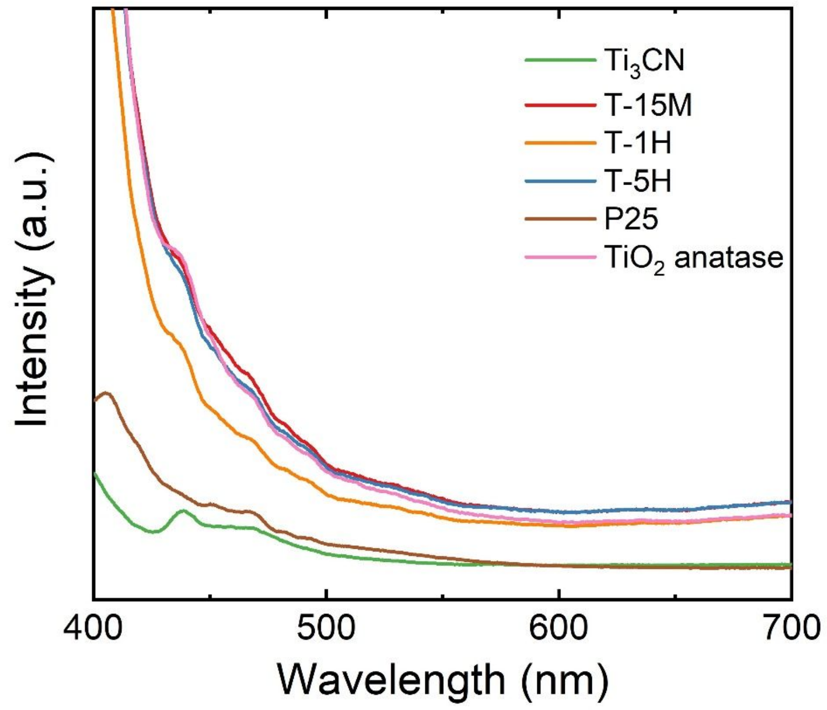

Figure S12. PL spectra of Ti_3CN, T-15M, T-1H, and T-5H compared with the reference TiO_2 anatase (sol-gel) and P25.

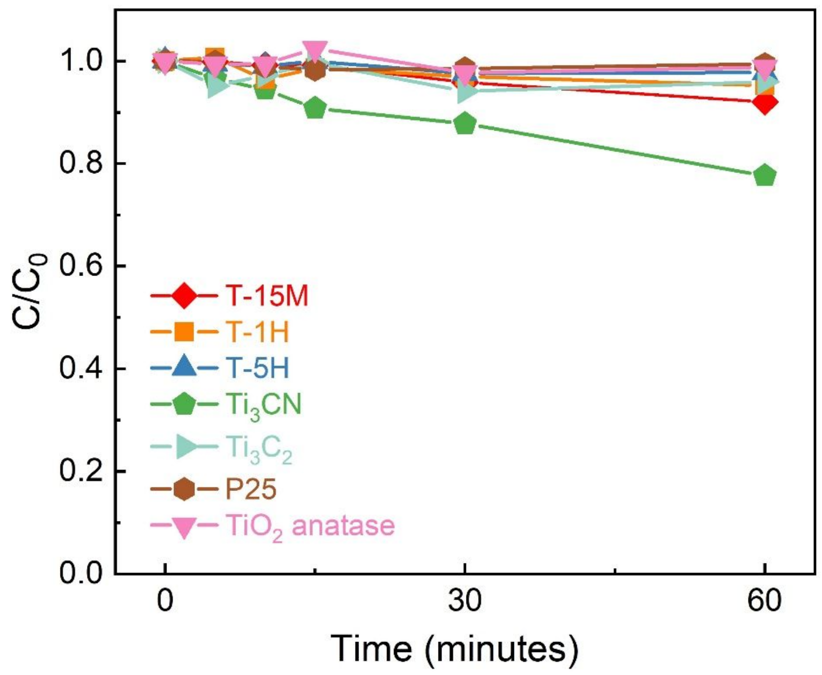

Figure S13. Adsorption of methylene blue on the sample in the dark condition.

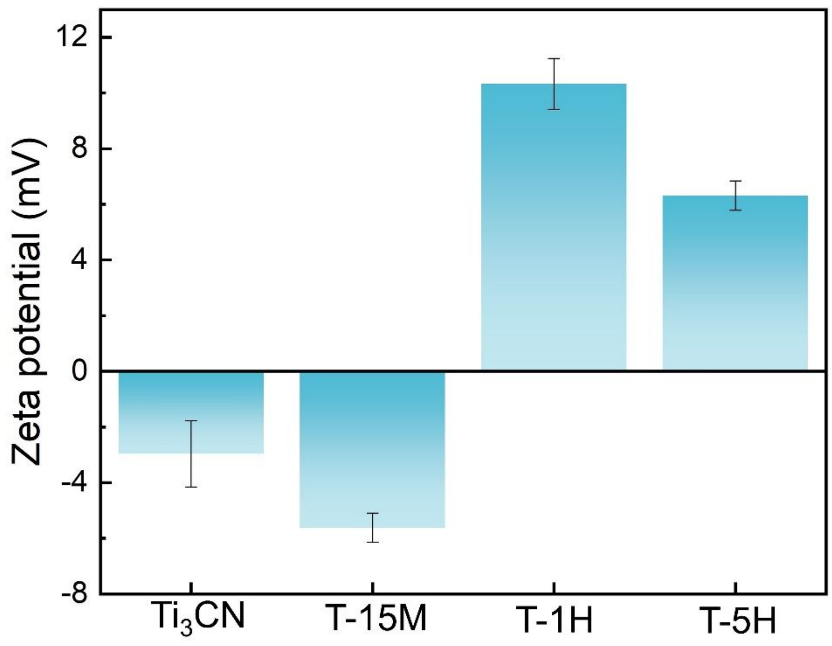

Figure S14. Zeta potential of Ti_3CN, T-15M, T-1H, and T-5H.

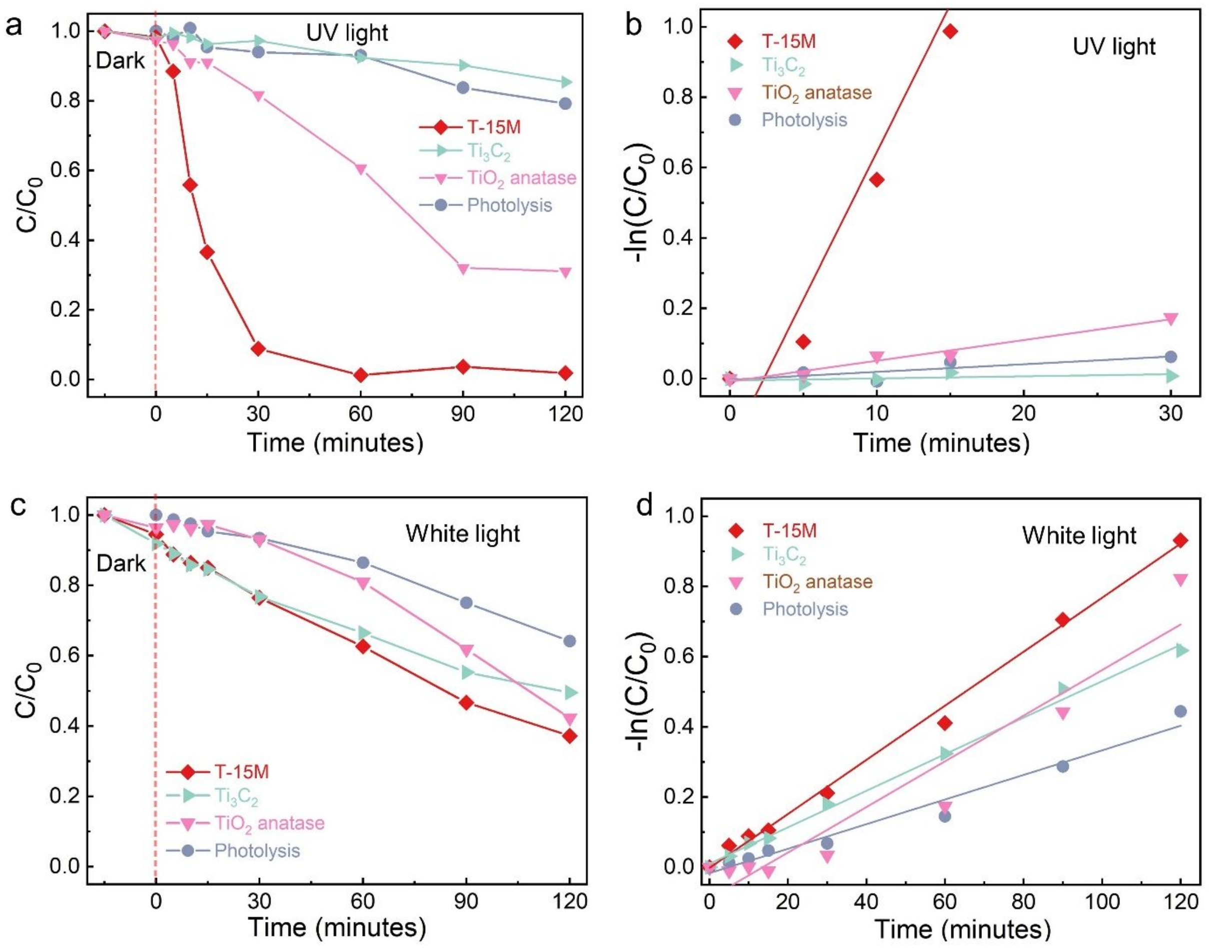

Figure S15. (a) Decomposition and (b) kinetic of the methylene blue decomposition in the presence of T-15M, Ti_3C_2 and TiO_2 anatase under UV light. (c) decomposition and (d) kinetic under simulated white light irradiation. The decomposition without the catalysts (photolysis) is also plotted in the graph.

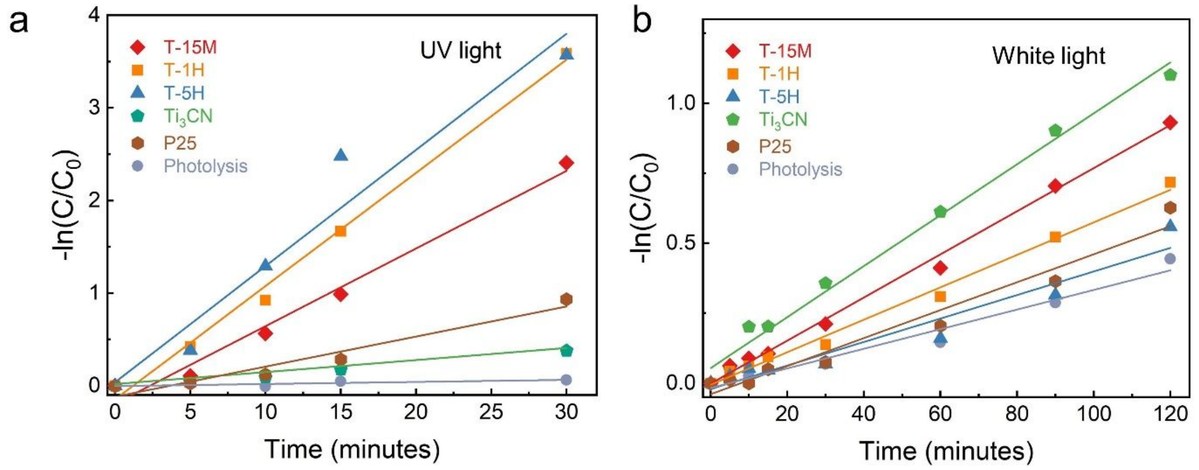

Figure S16. (a) Kinetic of methylene blue decomposition under (a) UV light and (b) simulated white light irradiation in the presence of Ti_3CN, T-15M, T-1H, T-5H, and P25. The decomposition without the catalysts (photolysis) is also plotted in the graph.

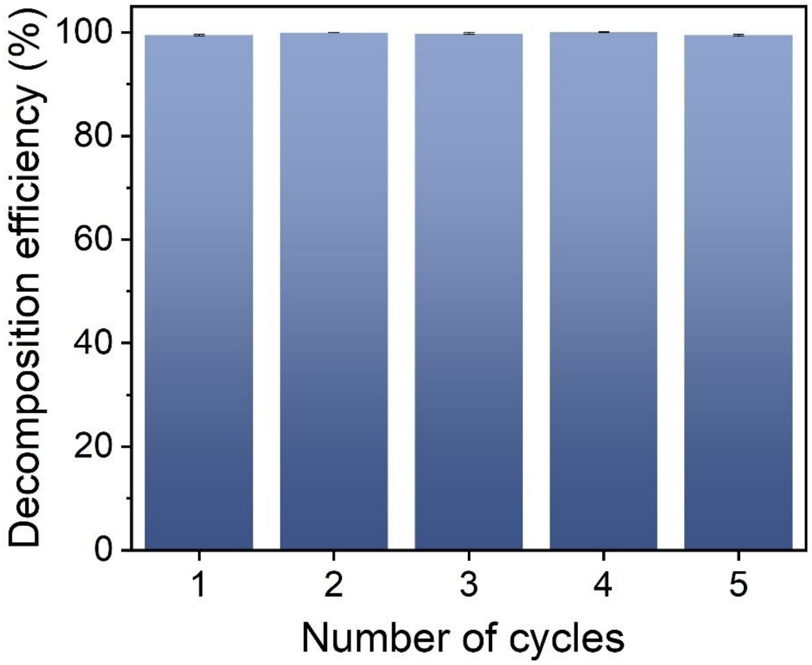

Figure S17. Reusability of T-15M in five subsequent photocatalytic cycles towards methylene blue.

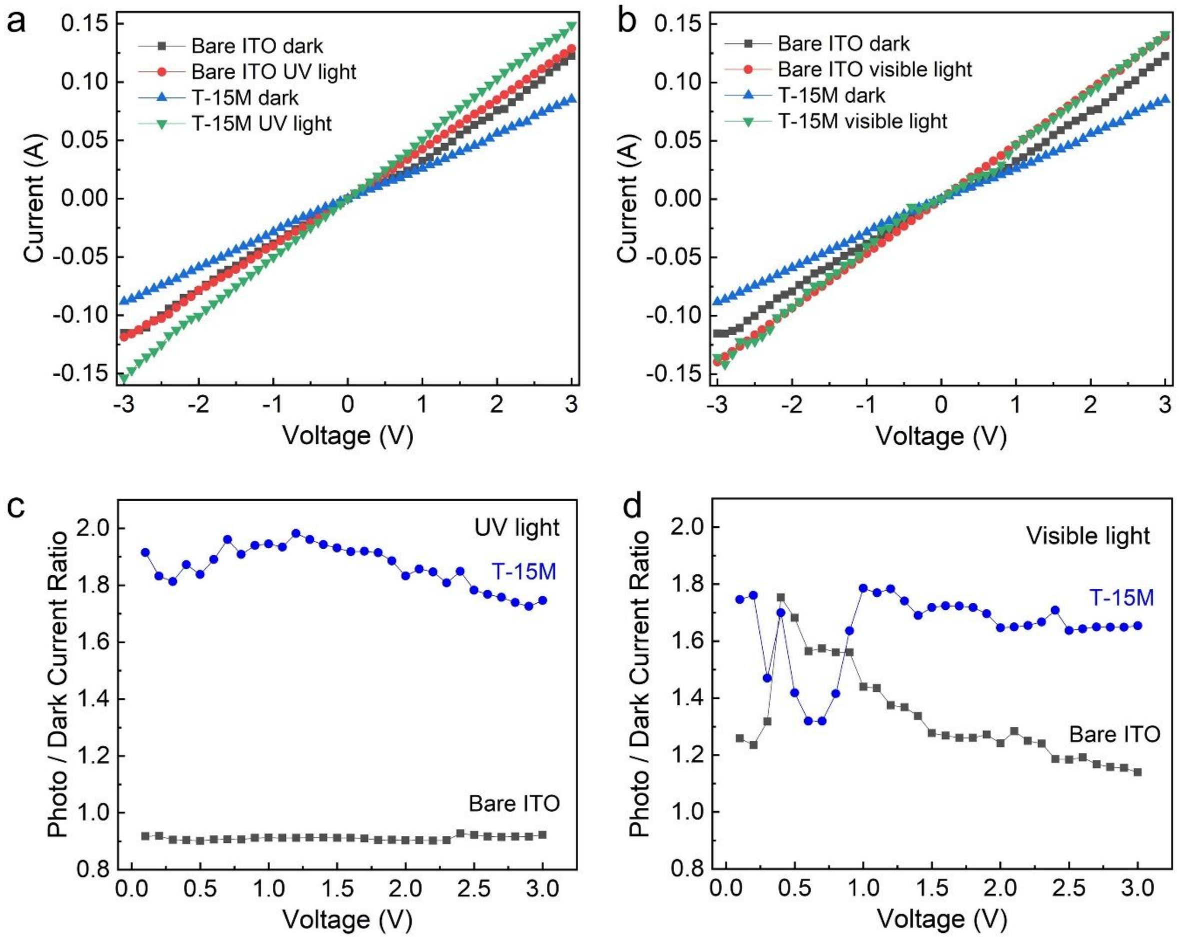

Figure S18. The current-voltage curve of bare ITO and T-15M under (a) visible and (b) UV light irradiation. Photo-to-dark current ratio of bare ITO and T-15M under (c) visible and (d) UV light irradiation.

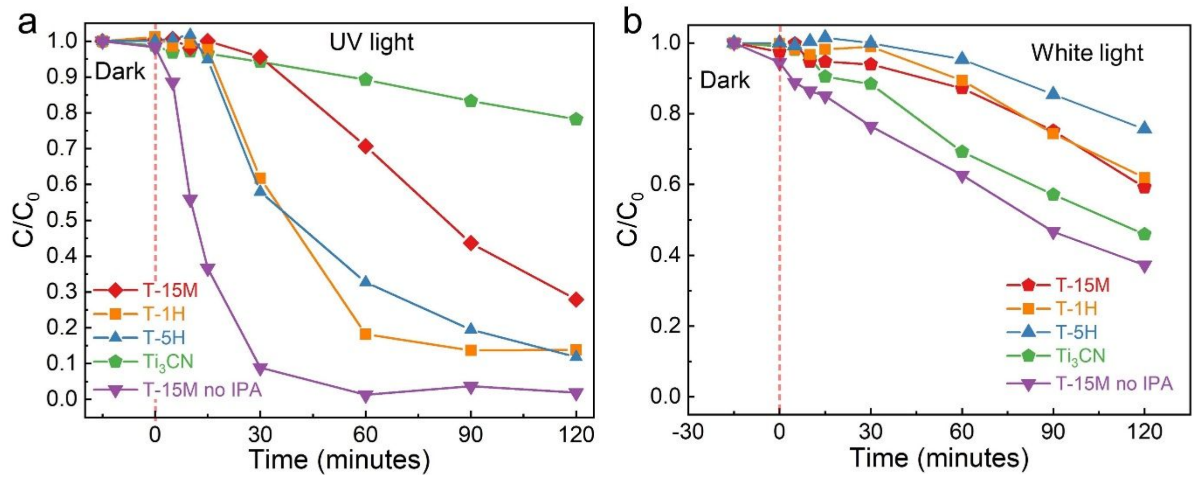

Figure S19. Photocatalytic MB decomposition with the presence of isopropanol as •OH scavenger under (a) UV and (b) simulated white light.

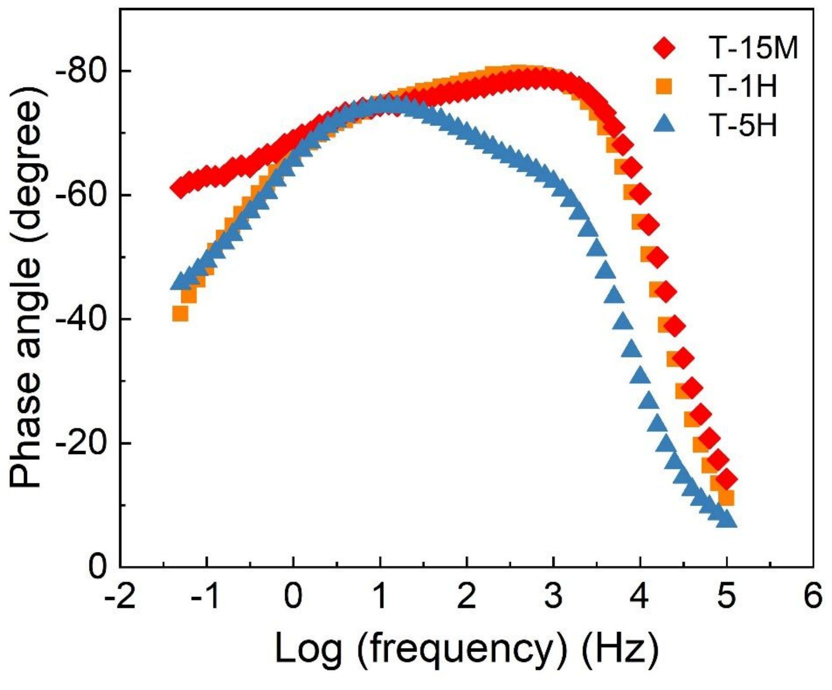

Figure S20. Bode EIS plots of T-15M, T-1H, and T-5H.

Table S1. Rate constant of MB decomposition in the presence of photocatalysts under UV and simulated white light irradiation.

Catalyst used in the process	Rate constant (k) × 10^{-3} (min^{-1})	
	UV light	White light
Photolysis	2.2	3.5
Ti_3CN	13.0	9.1
T-15M	83.8	7.7
T-1H	122.3	5.8
T-5H	125.4	4.2
P25	32.5	5.0
TiO_2 anatase	5.9	6.5
Ti_3C_2	0.6	5.2

Table S2. Comparison of photocatalytic performance for dye degradation between our work and previously reported TiO_2-based photocatalysts.

Catalyst	Synthesis method	Amount of catalyst per 1 mL process (mg)	Decomposed dye	Dye concentration (mg/L)	Process time (min)	Process efficiency	Kinetics (min^{-1})	Ref.
TiO_2	Hydrothermal	0.1	Methylene blue	8	180	90% (UV)	0.012	[6]
TiO_2	Sol-gel	0.2	Methylene blue	10	180	82% (UV)	0.010	[7]
TiO_2 Degussa P25 nanoparticles	Focused ultrasonic dispersion	0.1	Methylene blue	10	60	86% (UV)	-	[8]
Ti_3C_2-derived TiO_2	Auto-oxidation in DI water	0.4	Methylene blue	10	60	40% (UV)	0.007	[9]
TiO_2/Ti_3C_2	Solvothermal	1	Methyl orange	10	75	91% (UV)	0.015	[10]
TiO_2/Ti_3C_2	Hydrothermal	0.5	Methylene blue	60	60	96% (UV)	-	[11]
TiO_2/Ti_3C_2	Hydrothermal	0.1	Rhodamine B	5	30	40% (Solar simulator)	-	[12]
(001) TiO/Ti_3C_2	Hydrothermal	0.05	Methyl orange	20	50	97% (UV)	-	[13]
TiO_2/Ti_3C_2	Hydrothermal	0.4	Rhodamine B	10	40	97% (Solar simulator)	-	[14]
TiO_2/Ti_3C_2	Electrostatic self-assembly	1	Methyl orange	20	40	99% (UV)	0.102	[15]
Ti_3C_2-derived carbon-supported TiO_2	Thermal annealing	0.2	Methylene blue	20	60	99% (UV)	-	[16]
Truncated bipyramid TiO_2/Ti_3CN	Microwave hydrothermal	0.5	Methylene blue	25	30	98% (UV)	0.125	our work

Table S3. EIS fitting parameters of Ti_3CN, T-15M, T-1H, and T-5H.

Sample	Parameter	Value	Unit
Ti_3CN	R_{el}	14.87	Ohm
	Q_1	22.64×10^{-6}	F
	A_1	0.73	Constant
	R_t	1269	Ohm
	Q_2	36.55×10^{-6}	F
	A_2	0.69	Constant
	R_{ct}	207.16	kOhm
T-15M	R_{el}	13.66	Ohm
	Q_1	1.07×10^{-6}	F
	A_1	0.94	Constant
	R_t	2281	Ohm
	Q_2	2.26×10^{-6}	F
	A_2	0.61	Constant
	R_{ct}	25794.94	kOhm
T-1H	R_{el}	15.89	Ohm
	Q_1	1.33×10^{-6}	F
	A_1	0.92	Constant
	R_t	12162	Ohm
	Q_2	1.70×10^{-6}	F
	A_2	0.56	Constant
	R_{ct}	1508.99	kOhm
T-5H	R_{el}	14.67	Ohm
	Q_1	1.15×10^{-6}	F
	A_1	0.81	Constant
	R_t	146493	Ohm
	Q_2	33.32×10^{-6}	F
	A_2	1	Constant
	R_{ct}	185.73	kOhm

Legend:

- R_{el} = Resistance of electrolyte.
- Q_1 = Capacitance of substrate/electrolyte interface.
- Q_3 = Capacitance of charge storage in TiO_2 (Ti_3CN) coated GCE electrode.
- A = Constant phase element (CPE) exponent that characterizes the phase shift.
- R_t = Transport resistance of TiO_2 (Ti_3CN)
- R_{ct} = Charge transfer resistance at the TiO_2 (Ti_3CN)/electrolyte interface.

References

(1) Dulian, P.; Nachit, W.; Jaglarz, J.; Zięba, P.; Kanak, J.; Żukowski, W. Photocatalytic Methylene Blue Degradation on Multilayer Transparent TiO_2 Coatings. *Opt. Mater.* **2019**, *90*, 264–272. https://doi.org/10.1016/j.optmat.2019.02.041.

(2) Krishnamoorthy, K.; Mohan, R.; Kim, S.-J. Graphene Oxide as a Photocatalytic Material. *Appl. Phys. Lett.* **2011**, *98* (24), 244101. https://doi.org/10.1063/1.3599453.

(3) Williams, G.; Seger, B.; Kamat, P. V. TiO_2-Graphene Nanocomposites. UV-Assisted Photocatalytic Reduction of Graphene Oxide. *ACS Nano* **2008**, *2* (7), 1487–1491. https://doi.org/10.1021/nn800251f.

(4) Liu, Y.; Liu, X.; Dong, W.; Zhang, L.; Kong, Q.; Wang, W. Efficient Adsorption of Sulfamethazine onto Modified Activated Carbon: A Plausible Adsorption Mechanism. *Sci. Rep.* **2017**, *7* (1), 12437. https://doi.org/10.1038/s41598-017-12805-6.

(5) Cui, Y.; Ding, Z.; Liu, P.; Antonietti, M.; Fu, X.; Wang, X. Metal-Free Activation of H_2O_2 by g-C_3N_4 under Visible Light Irradiation for the Degradation of Organic Pollutants. *Phys. Chem. Chem. Phys.* **2012**, *14* (4), 1455–1462. https://doi.org/10.1039/C1CP22820J.

(6) Nguyen-Phan, T.-D.; Shin, E. W. Morphological Effect of TiO_2 Catalysts on Photocatalytic Degradation of Methylene Blue. *J. Ind. Eng. Chem.* **2011**, *17* (3), 397–400. https://doi.org/10.1016/j.jiec.2011.05.013.

(7) Hwang, K.-J.; Lee, J.-W.; Shim, W.-G.; Jang, H. D.; Lee, S.-I.; Yoo, S.-J. Adsorption and Photocatalysis of Nanocrystalline TiO_2 Particles Prepared by Sol–Gel Method for Methylene Blue Degradation. *Adv. Powder Technol.* **2012**, *23* (3), 414–418. https://doi.org/10.1016/j.apt.2011.05.010.

(8) Kim, S.-Y.; Lee, T.-G.; Hwangbo, S.-A.; Jeong, J.-R. Effect of the TiO_2 Colloidal Size Distribution on the Degradation of Methylene Blue. *Nanomaterials* **2023**, *13* (2). https://doi.org/10.3390/nano13020302.

(9) Kumar, S.; Park, H. M.; Nguyen, V. H.; Kim, M.; Nasir, N.; Suleman, M.; Lee, S.; Seo, Y. Oxidation-Driven Auto-Conversion of $Ti_3C_2T_x$ MXene to TiO_2 Nanoparticles for Photocatalytic Applications. *J. Alloys Compd.* **2024**, *976*, 173399. https://doi.org/10.1016/j.jallcom.2023.173399.

(10) Chen, J.; Zheng, H.; Zhao, Y.; Que, M.; Wang, W.; Lei, X. Morphology and Photocatalytic Activity of TiO_2/MXene Composites by in-Situ Solvothermal Method. *Ceram. Int.* **2020**, *46* (12), 20088–20096. https://doi.org/10.1016/j.ceramint.2020.05.083.

(11) Zhang, S.; Cai, M.; Wu, J.; Wang, Z.; Lu, X.; Li, K.; Lee, J.-M.; Min, Y. Photocatalytic Degradation of TiO_2 via Incorporating Ti_3C_2 MXene for Methylene Blue Removal from Water. *Catal. Commun.* **2023**, *174*, 106594. https://doi.org/10.1016/j.catcom.2022.106594.

(12) Lee, D.-E.; Mameda, N.; Devthade, V.; Jo, W.-K.; Tonda, S. A Hierarchical 3D Hybrid CoAl-Layered Double Hydroxide/TiO_2/Ti_3C_2 MXene S-Scheme Photocatalyst with 2D/0D/2D Contact Interfaces for Sustainable Pollutant Degradation. *Appl. Surf. Sci.* **2024**, *649*, 159178. https://doi.org/10.1016/j.apsusc.2023.159178.

(13) Peng, C.; Yang, X.; Li, Y.; Yu, H.; Wang, H.; Peng, F. Hybrids of Two-Dimensional Ti_3C_2 and TiO_2 Exposing {001} Facets toward Enhanced Photocatalytic Activity. *ACS Appl. Mater. Interfaces* **2016**, *8* (9), 6051–6060. https://doi.org/10.1021/acsami.5b11973.

(14) Quyen, V. T.; Ha, L. T. T.; Thanh, D. M.; Le, Q. V.; Viet, N. M.; Nham, N. T.; Thang, P. Q. Advanced Synthesis of MXene-Derived Nanoflower-Shaped TiO_2@Ti_3C_2 Heterojunction to Enhance Photocatalytic Degradation of Rhodamine B. *Environ. Technol. Innov.* **2021**, *21*, 101286. https://doi.org/10.1016/j.eti.2020.101286.

(15) Li, H.; Sun, B.; Gao, T.; Li, H.; Ren, Y.; Zhou, G. Ti_3C_2 MXene Co-Catalyst Assembled with Mesoporous TiO_2 for Boosting Photocatalytic Activity of Methyl Orange Degradation and Hydrogen Production. *Chinese J. Catal.* **2022**, *43* (2), 461–471. https://doi.org/10.1016/S1872-2067(21)63915-3.

(16) Rajavel, K.; Shen, S.; Ke, T.; Lin, D. Photocatalytic and Bactericidal Properties of MXene-Derived Graphitic Carbon-Supported TiO_2 Nanoparticles. *Appl. Surf. Sci.* **2021**, *538*, 148083. https://doi.org/10.1016/j.apsusc.2020.148083.
